# Measuring multisector nutrition and health intervention coverage using composite coverage analysis methods: a scoping review and methodological guidance

**DOI:** 10.1136/bmjopen-2025-111298

**Published:** 2026-06-17

**Authors:** Taylor Morrison, Nadia Akseer, Rebecca Heidkamp, Abdoulaye Maïga, Hana Tasic

**Affiliations:** 1 Department of International Health, Johns Hopkins University Bloomberg School of Public Health, Baltimore, Maryland, USA; 2 Modern Scientist Global, St Catharines, Ontario, Canada

**Keywords:** Health Equity, Health Services Accessibility, Health policy

## Abstract

**Abstract:**

**Objectives:**

Composite indices provide an opportunity to measure the reach of multisector strategies as countries progress towards Sustainable Development Goals and the achievement of nutrition, health and other sectoral targets. Composite measures allow for the measurement of nutrition and health intervention coverage across sectors and can be used to benchmark progress, track nutrition goals and assess inequities of multisector nutrition interventions. Of the many composite indices in use to measure health and nutrition intervention coverage and population status as a means of assessing progress in achieving health and nutrition targets, few have been previously documented in the nutrition and health literature, nor have these indicators been described in detail. This scoping review aimed to identify composite coverage indices that capture nutrition and health intervention coverage or status, summarise their estimation methodologies and validation approaches and evaluate their strengths and weaknesses.

**Design:**

Scoping review conducted in accordance with the Joanna Briggs Institute Reviewer Manual following the Preferred Reporting Items for Systematic Reviews and Meta-Analyses Extension for Scoping Reviews (PRISMA-ScR) verification list.

**Data sources:**

PubMed, EMBASE, Scopus, Google Scholar and Global Health registries were searched from 1980 to 3 March 2026.

**Eligibility criteria:**

We included studies of any design that described, used or developed a composite measurement of health or nutrition intervention coverage or status.

**Data extraction and synthesis:**

One reviewer screened the titles and abstracts and full text using Covidence and abstracted data from each article using Microsoft Excel using standardised methods.

**Results:**

We retrieved and screened a total of 7120 records of which 154 articles were included. We identified a total of 56 unique indices (25 universal healthcare coverage; 14 reproductive, maternal, newborn, child health (RMNCH) coverage; 10 nutritional status, food security or nutrition intervention coverage; 6 health service coverage and 1 combined universal healthcare, health risk factors and nutrition coverage measure). We identified three major formula construction methodologies: normative (n=34), statistical (n=13) and participatory (n=10). Together, the indices employed six different aggregation methods: weighted linear mean (n=28), geometric mean (n=13), linear mean (n=9), random-effects meta-analysis (n=6), summation (n=2) and weighted geometric mean (n=1). More than one third of the indices identified have not been validated in the literature (n=21).

**Conclusions:**

Our review identified a significant gap in composite nutrition intervention coverage index availability, methodological frameworks for index design and index validation. There is a need for additional resources for guiding policy and programme actors to develop validated, fit for purpose composite nutrition-specific and nutrition-sensitive coverage indices. We propose that a framework be developed for stakeholders to guide composite index construction for multisectoral nutrition intervention coverage measurement.

**STRENGTHS AND LIMITATIONS OF THIS STUDY**

- This scoping review used a systematic search strategy with supplementary searching using snowballing, which compiled a diverse array of studies from multiple disciplines and included grey literature.
- One review author screened and extracted the data with ambiguities collaboratively addressed in team meetings.
- The Joanna Briggs Institute Reviewer Manual following the Preferred Reporting Items for Systematic Reviews and Meta-Analyses Extension for Scoping Reviews (PRISMA-ScR) verification list was used to conduct this scoping review.

## Introduction

 Measuring individuals being reached with nutrition interventions is a common priority as countries progress towards Sustainable Development Goals (SDGs)—17 goals adopted by the United Nations to address global challenges to sustainable development—for women’s and children’s health and nutrition.^1^ Evidence from Exemplars in Global Health (EGH) research—an initiative from EGH to identify and analyse countries achieving outstanding public health progress to understand what makes them successful—shows that reaching women, children and households with multiple effective interventions across sectors is critical for achieving nutrition targets.^2 3^ Many low- and middle-income countries have multisector strategies in place to address malnutrition that combine health, food, social protection and other interventions within and across key populations. For example, micronutrient control strategies combine micronutrients delivered through prenatal supplements and large-scale food fortification, with social and behavioural change communication and other interventions to improve dietary diversity. Measuring who is being reached is the first step to developing models that estimate the contribution of various interventions on key health outcomes. It can therefore assist in developing and measuring the reach of multisectoral strategies to control nutrition problems; estimating intake, sufficiency and excess; and monitoring the scaling back of interventions in the transition of intervention strategies. However, monitoring implementation of these multisector and multi-intervention strategies can be a challenge as (1) there is no standard approach to estimating layered coverage of multiple interventions, (2) methods used to assess reach or coverage vary across sectors and delivery platforms (eg, household survey, administrative data) and (3) it is rare that a single data source includes population-based coverage estimates for all interventions.

Composite indices are summary measures, aggregated at the group-level that bring together discordant data sources to estimate an overall outcome of interest.^4^ While individual measures of health and nutrition intervention coverage are useful in assessing the progress of specific interventions, composite measures allow stakeholders to measure the comprehensiveness of nutrition and health interventions across sectors. These composite summary measures can be used to benchmark progress, track nutrition goals and assess inequities of multisector nutrition interventions. They also simplify cross-country and cross-time comparisons by using a single measure comprised of a standardised set of indicators. The layered, population-level coverage of interventions is particularly important for multisector nutrition strategies which assume that vulnerable groups are being reached by multiple interventions across sectors to achieve nutrition goals. Composite indices are widely used in economics and social development literature—such as the Human Development Index (HDI)^5^—and more recently, for health and nutrition applications with varied adoption. There are several composite indices that measure the layered coverage of health and nutrition services and/or health and nutritional status at the population level, including the Composite Coverage Index (CCI)—formerly the Coverage Gap Index (CGI)^4 6^—and Universal Health Coverage Service Coverage Index (UHC SCI).^7^

Data for Decisions in Nutrition (DataDENT)^8^ aims to develop flexible methods to estimate the layered coverage of multiple interventions across sectors and key populations for the purpose of policy design and monitoring. Our goal is to develop a stepwise framework for designing composite coverage indices that are fit for purpose of nutrition policy and programme actors. As a first step, we carried out a scoping review to identify different approaches to developing composite indices capturing coverage of multiple health and/or nutrition interventions or health and nutritional status. The aims of the scoping review were (1) to identify composite coverage indices that capture health and nutrition intervention coverage or status; (2) to summarise the general methodologies of composite coverage estimation and their validation approaches; and (3) to evaluate the strengths and weaknesses of various composite coverage estimation methodologies.

## Methods

This scoping review was designed and conducted in accordance with the Joanna Briggs Institute Reviewer Manual^9^ following the Preferred Reporting Items for Systematic Reviews and Meta-Analyses Extension for Scoping Reviews (PRISMA-ScR) verification list.^10^ The protocol for this scoping review can be found in the online supplemental material—protocol.

### Database selection and search strategy

We searched the following electronic bibliographic databases for published and grey literature: PubMed, EMBASE (Ovid), Scopus, Google Scholar and Global Health (Global Health, 1910 Forward; OvidSP). The first 10 pages of results from Google Scholar were extracted. Supplementary searching, using snowballing, was used to identify other relevant published and grey literature.

The search strategy included terms related to composite measurement, methods-based research and health-specific and nutrition-specific interventions. Specific keywords identified in the preliminary searches were added to the final search strategy. The search strategy for EMBASE was modified as appropriate for each database (online supplemental material—protocol).

One author (TM) developed the search strategy. All three authors were included in the review and approval of the search strategy. The search was conducted on 18 May 2024, by one author (TM). An updated search was conducted on 3 March 2026, by one author (TM). Search results were imported to Covidence and duplicates were removed. The authors (TM and NA) developed criteria for level 1 and 2 assessments based on the inclusion and exclusion criteria. Citation abstracts and full text articles were uploaded to Covidence for screening and further review.

Studies of any design that included composite measurement of health or nutrition intervention coverage or status were eligible for inclusion. Publications describing, assessing or using composite analysis methods were eligible for inclusion. Composite measurement was defined as any aggregate formula that uses group-level variables for imputation that represents the average health and nutrition intervention coverage and/or nutritional status of a population (ie, population-level). We applied criteria to exclude co-coverage measurements (ie, individual-level) in line with the specifications set out by Wehrmeister *et al*.^4^ Measures of individual-level health and nutrition status or intervention coverage were excluded. Measures of disease burden and health impact, such as health-adjusted life years, quality-adjusted life years and disability-adjusted life years, were excluded. These measures of relative disease burden and health impact were excluded as they do not measure health or nutrition intervention coverage or status. All populations and settings were considered. We extracted indicators, scoring methods and analytical approaches as reported in publications. We included both published and grey literature reported in the English language. Electronic databases, contact with study authors, trial registers, grey literature obtained or published between 1980 and 3 March 2026, were considered. The limit of 1980 was selected with the intention of including literature on foundational composite indices, such as the HDI which was introduced in 1990 and is universally accepted and used. We therefore assumed that there could be methodological papers published between 1980 and 1990 that described the development of similar composite indices used to measure nutrition and health intervention coverage or status.

One reviewer (TM) independently screened the titles and abstracts yielded by the search against the inclusion and exclusion criteria. Full reports were obtained for titles that appeared to meet the inclusion criteria. The reviewer (TM) then screened full text reports and decided whether they met the inclusion criteria. The reviewer (TM) recorded reasons for excluding reports.

Standardised forms for data extraction were piloted and tested. Extracted data and information from each eligible source were confirmed by the reviewer (TM). The reviewer (TM) resolved uncertainties by discussion with the coauthors (NA and RH). Data abstracted included geographical application, composite measure name, domain of measurement, datasets used for estimation, formula construction methodology, aggregation methodology, scoring formula, indicator selection criteria or methods, indicators included in the formula, index disaggregation and validation methods.

We chose to categorise the composite indices identified in this review by the domain of measurement (health services, nutrition, universal healthcare and reproductive, maternal, newborn and child health (RMNCH)). We categorised the identified formula construction and aggregation methodologies following the Handbook of Constructing Composite Indicators published by the Organisation for Economic Co-operation and Development (OECD; the Statistics Directorate and the Directorate for Science, Technology and Industry) and the Econometrics and Applied Statistics Unit of the Joint Research Centre (JRC) of the European Commission.^11^ We categorised formula construction methodologies as statistical, normative or participatory.^11^ Statistical approaches for grouping indicators include principal component analysis (PCA), factor analysis (FA) and reliability or item analysis. Normative approaches to formula construction encompass approaches that are based on standards of practice or programming. For example, indicators may be selected and organised based on the life stage during which they are delivered, data availability, association with the nutrition outcome of interest, policy contexts and goals or programme availability and importance. Normative weighting may also be based on the number of contacts or doses required for adequate intervention coverage, intervention impact on nutritional status, potential health gains of the intervention, health expenditure shares or contribution to policy goals. Equal weighting is also included in the normative category due to the underlying assumptions that equal weighting imposes. Importantly, equal weighting may indicate the omission of empirical methods or the derivation of equal weighting using normative methods described above if not described otherwise. Participatory approaches to formula construction include expert and public opinion. This method can be applied non-systematically, through expert consensus or systematically, through approaches such as the analytic hierarchy process (AHP; including fuzzy AHP (FAHP)) and Delphi methodology.^11^ Aggregation methods were similarly defined as linear or geometric following the Handbook of Constructing Composite Indicators published by the OECD and JRC of the European Commission.^11^ Linear aggregation is defined by use of the arithmetic mean to aggregate indicators to calculate the index. Geometric aggregation is defined by use of the geometric mean to aggregate indicators to calculate the index. We further categorised aggregation methods as weighted and unweighted linear or geometric means. Unlike the OECD, we used linear and arithmetic aggregation interchangeably. Additionally, without guidance on categorisation of validation methods, we grouped validation methods identified using the language established in the literature reviewed.

### Patient and public involvement

Due to the nature of this scoping review, it was not appropriate to involve members of the public in the design, conduct, reporting or dissemination plans of the research.

## Results

### Selection of sources of evidence

**Figure 1:**
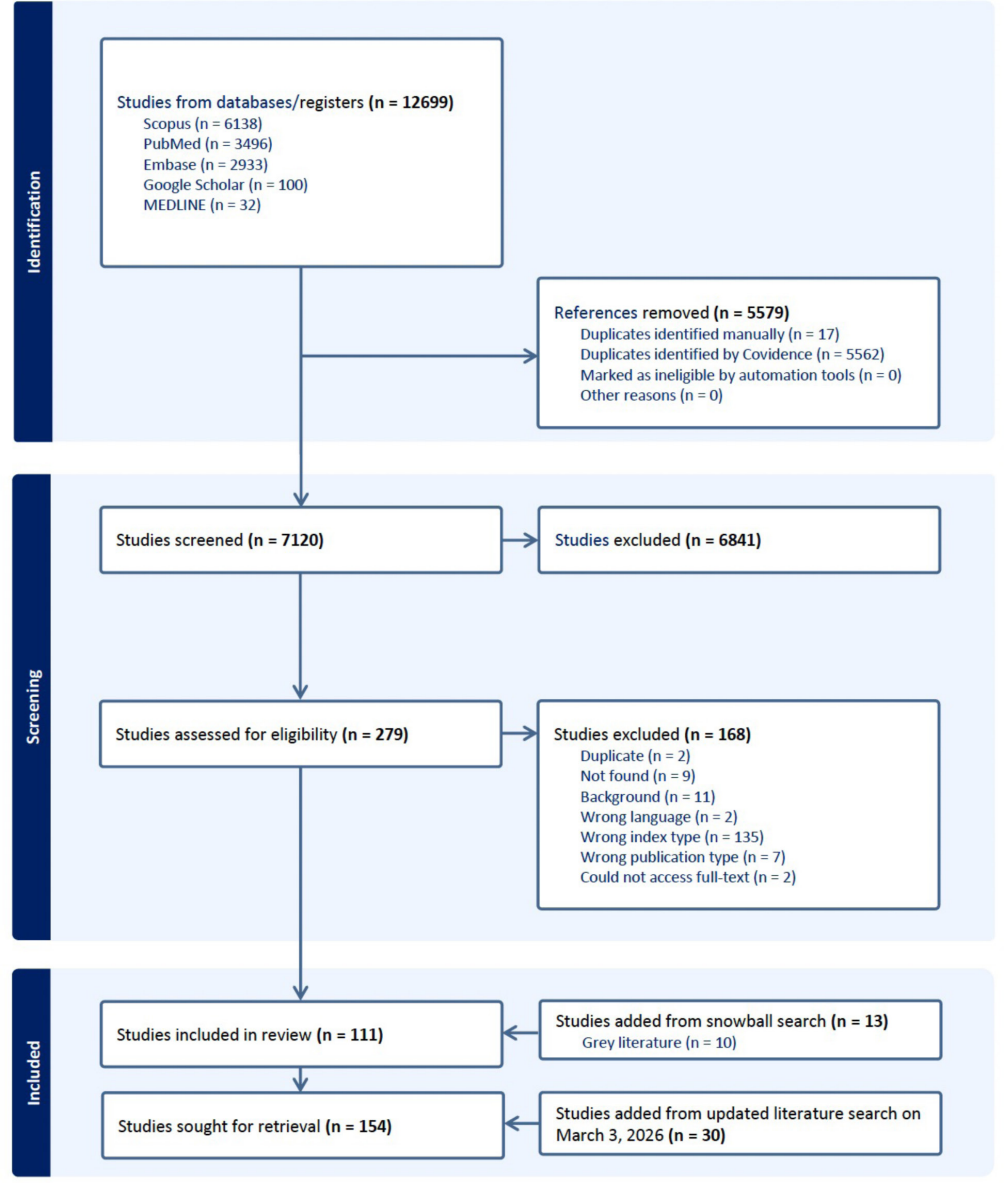
Preferred Reporting Items for Systematic Reviews and Meta-Analyses flow diagram depicting results of the literature search and review procedure.

A total of 12 699 records were retrieved from database searches (figure 1). After removing duplicates, 7120 abstracts were screened for eligibility and subsequently, 279 full-text articles were assessed. An additional 30 articles were retrieved from an updated literature search on 3 March 2026. An additional 13 articles were retrieved from snowballing the search with included articles. Of the 13 articles retrieved from snowballing, 10 were grey literature sources. A final 154 articles were included.

A total of 154 studies were included that described, analysed or applied composite indices related to health and nutrition intervention coverage or status. Notably, a number of studies simultaneously described index development, analysis and application. Of the 154 publications, the majority described the application (n=122) and the development of composite indices (n=59). Additionally, there were a small number of studies that described the analysis of indices (n=14). A total of 56 unique indices were identified from the 154 included studies (online supplemental table 1). Of the 56 unique indices, 25 measure universal healthcare coverage; 14 measure RMNCH coverage; 10 measure nutritional status, food security or nutrition intervention coverage (of which only 1 measures nutrition intervention coverage); 6 measure health service coverage; and 1 measures universal healthcare, health risk factors and nutrition in combination. Four of the 10 grey literature sources described, revised or used the Global Hunger Index, formerly the International Nutrition Index (NI).^12^,^15^ The other six grey literature sources are WHO reports of the Universal Health Coverage Service Coverage Index (UHC SCI).^16^,^21^

A total of 34 indices were constructed using a normative approach (table 1). Under the normative approach, equal weighting (n=16); weighting reflecting the contribution of each indicator to the measured outcome (n=8); intervention standards (number of contacts or doses), interventions implemented and data available (n=7); and weighting reflecting inputs (health expenditure), norms and policy priorities (n=3) were applied. A total of 13 indices applied statistical formula construction methods, which included PCA (n=8), FA (n=2) and hybrid double reference point and Pena’s distance P2 (n=3) approaches. Of the 10 indices using participatory approaches, indices applied expert consensus or public opinion approaches using non-systematic methods (n=7), AHPs (AHP or FAHP) (n=2) and the Delphi method (n=1) for formula construction. Notably, one study applied both normative (equal weighting) and statistical (PCA) approaches to the Composite Index of Health^22^ and is therefore counted twice.

**Table 1:** Formula construction methodologies identified

Formula construction methodology (number of indices)	Types of formula construction methods	Number of indices, n
Normative (n=34)	Equal weighting	16^*a*^
	Intervention standards (number of contacts or doses), interventions implemented and data available	7
	Weighting reflects contribution of each indicator to the measured outcome	8
	Weighting reflects inputs (health expenditure), norms and policy priorities	3
Statistical (n=13)	PCA	8^*a*^
	Factor analysis	2
	Hybrid double reference point and Pena’s distance P2	3
Participatory (n=10)	Expert consensus or public opinion (non-systematic)	7
	Analytical hierarchy process (including fuzzy analytical hierarchy process)	2
	Delphi method	1

The Composite Index of Health had both normative equal weighting and statistical PCA applied to the construction of the composite measure and is therefore reflected in this table twice.^22^

PCA, principal component analysis.

Importantly, a methodological overlap exists between participatory and normative approaches since expert consensus is generally required to establish norms of health and nutrition measurement. For example, the CCI is generally considered a normative approach that is based on the interventions that comprise the continuity of care and the number of contacts or doses required for a particular intervention. However, the CCI was first developed as the CGI, by which composite indicators included in the index were selected based on several criteria defined and evaluated by authors and experts in the field.^6^

**Figure 2:**
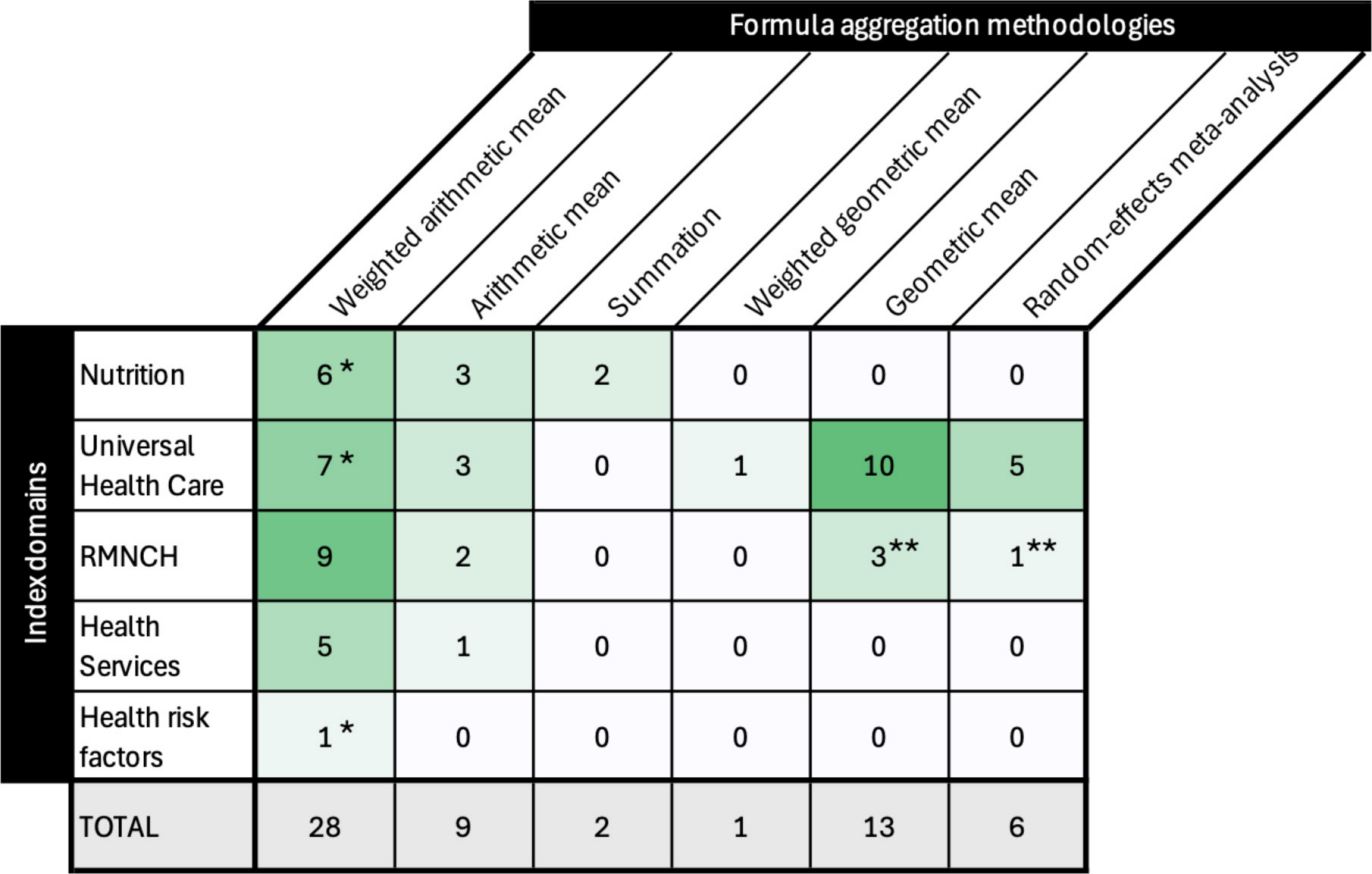
Heat map of aggregation methodologies identified by index domain of measurement (n=56). *The composite HRFI and subindices (NUTI, ERFI and NCDI) measure three domains—nutrition, universal health care and health risk factors—and is therefore represented three times in Figure 2. **The RMNCH Health Service Coverage and subindices (prevention and treatment subindices) are aggregated using geometric mean and random-effects meta-analysis, respectively. The index is therefore represented two times in Figure 2. ERFI, Environmental Risk Factors Index; HRFI, Health Risk Factor Index; NCDI, Non-communicable Disease Index; NUTI, Nutrition Index; RMNCH, reproductive, maternal, newborn and child health.

Weighted linear mean was the primary aggregation method identified among the composite indices included (n=28) (figure 2). Geometric mean (n=13), linear mean (n=9), random-effects meta-analysis (n=6), summation (n=2) and weighted geometric mean (n=1) were also identified as aggregation methods. The primary aggregation methods used in composite indicator development were linear (arithmetic) and geometric means. Arithmetic means bring together indicators through addition and multiplication. Geometric means bring together indicators using the product and the *n*th root of the values.

For more than one third of the indices identified we did not find documentation of their validation (n=21). Among the indices that had validation methods applied to them, sensitivity analysis was the most common across publications (n=53) (table 2). This type of validation is conducted by assessing the difference between index scores using varying aggregation methods and/or indicator weights. Methods used in sensitivity analysis included rank correlation (eg, correlation between rank order of regions based on index estimates using different composite methods using Spearman rank correlation) (n=11), index correlation (eg, correlation coefficient between index estimates using different aggregation and/or indicator weighting) (n=10), absolute difference (eg, visually assessing the absolute difference between index estimates) (n=22) and reliability (eg, statistically measuring the consistency of the index estimates using different methodologies with Cronbach’s Coefficient alpha test) (n=10). Notably, while Cronbach’s Coefficient alpha (α) test is not a test of validity, this test of reliability is used to assess the internal consistency of the indicators applied in the calculation of the composite index and was therefore considered a sensitivity analysis for the purpose of this review. Another validation category identified in this review was methods that assessed the relationship of an index with key outcomes or indicators (n=24). This type of validation employs convergent validation (eg, the correlation between index estimates and health and nutrition outcomes of interest or potential correlated variables, such as under five mortality rate and stunting, and other validated health coverage outcomes, such as the CCI) (n=21), content validity (eg, an evaluation of how well an index relates to the indicators used to construct a measure of the outcome of interest, such as the percentage of 30 matrix cells of health service types against population-age groups covered by each index) (n=1), known groups (eg, index construct validation, such as testing the percentage of 16 country pairs correctly ranked based on comparative performance in an associated outcome of interest) (n=1), and receiver operating characteristic (ROC) curve analysis (eg, ROC curve of health or nutrition outcome and index estimate) (n=1). Cross-validation is considered a test of index scoring fit and indicator relevance and was used to validate indices (n=2) through leave-one-out cross-validation. Finally, agreement with reference standards was another category of validation identified (n=2). Agreement methods identified included agreement between index estimate and gold standard measure (Cohen’s kappa, n=1) and agreement on indicator weighting among experts (Krippendorff’s alpha, n=1).

**Table 2:** Validation methodologies used in the included studies

Validation category, n	Validation subcategory	Number of times validation type was applied
Relationship with key outcomes, 24	Convergent validity	21
	Content validity	1
	Known groups	1
	ROC curve analysis	1
Sensitivity analysis (ie, varying aggregation methods and indicator weights), 53	Rank correlation (Spearman rank correlation)	11
	Index correlation	10
	Absolute difference	22
	Reliability (Cronbach’s Coefficient alpha test)	10
Cross-validation, 2		2
Agreement with reference standards, 2	Cohen’s kappa	1
	Krippendorff’s alpha	1

Convergent validity refers to the correlation between index estimates and health and nutrition outcomes of interest or potential correlated variables (eg, under 5 mortality rate and stunting). Content validity refers to the evaluation of how well an index relates to the indicators used to construct a measure of the outcome of interest (eg, percentage of 30 matrix cells of health service types against population-age groups covered by each index). Cross-validation refers to the test of index scoring fit and relevance of the indicators (eg, leave-one-out cross-validation). Cohen’s kappa is the agreement between index estimate and gold standard measure. Krippendorff’s alpha is a measure of agreement on indicator weighting between experts.

Note: indices and studies may have used multiple validation methods of different types and of the same types.

ROC, receiver operating characteristic.

## Discussion

### Summary of evidence

This scoping review presents a summary of composite nutrition and health indices used and developed in the literature. We identified 56 unique composite indices, 3 categories of formula construction methodologies comprising 10 formula construction approaches, 6 formula aggregation methodologies and 4 categories of index validation methods comprising 11 validation techniques. To our knowledge, this is the first scoping review conducted to identify methodologies underlying composite health and nutrition coverage and status indices.

A scoping review was chosen in preference to a systematic review to ensure a broad scope of composite indices were captured and to include all study types and grey literature to identify all relevant indices to health and nutrition intervention coverage and status, which is a vast field. We understood that the field of multisectoral nutrition intervention coverage measurement was sparse and expected to review indices from other fields, such as health systems and economics, in which index utilisation is commonplace.

Our scoping review uncovered a significant gap in the literature on nutrition index availability relative to health indices, with only one nutrition intervention coverage index identified. Additionally, there are no existing methodological frameworks for index design and very few indices used have been validated. Therefore, additional resources are needed to support and expand multisectoral population-level nutrition intervention coverage measurement as a method of monitoring progress towards SDGs for women’s and children’s health and nutrition. Particularly, as we move to an increasingly intersectional approach to reaching women, children and households with multiple effective interventions across sectors. The need for composite nutrition indices is also drawn from evidence of the extensive use of the CCI and co-coverage index in the nutrition and health literature.^4 23 24^ The CCI is a weighted arithmetic mean of eight essential preventative and curative interventions categorised by intervention areas across the continuum of care (family planning, maternal and newborn care, child immunisation and case management of childhood illness).^4 23 24^ A normative approach was used to develop the CCI, previously the CGI, with a total of six potential intervention areas reviewed and indicators selected based on data availability, accuracy and consistency of measurement, relevance to health system strength and potential health gains.^4 6 23 24^ The utility of the index is underscored by the simple weighting and aggregation method, which provides adaptability and interpretability. The CCI is easily adapted to different contexts depending on the package of interventions offered in that geography (individual indicators can be removed or replaced as needed). The interpretation of the score as a measure of RMNCH coverage across the continuum of care is driven by the score’s use of sentinel indicators which are specific to the outcome of measure. Additionally, the CCI is represented by a percentage which is easy to communicate graphically, can be dichotomised or categorised ordinally and allows for uncertainty estimation. Importantly, the CCI has been validated by many researchers using a variety of methods.^425^,^32^ Despite this, there are important limitations to such indices including the potential for the composite measure to mask differential coverage between interventions, changes in survey methodology and in turn indicator definitions to bias results, and comparability across geographies to be limited by differences in intervention package availability. The CCI and co-coverage index are applied as companion aggregate measures of progress in the coverage of RMNCH interventions and serve as benchmarks for stakeholders in the field.^4 23 24^ However, the functional definitions of composite coverage and co-coverage are conflated in the literature and need to be clearly defined as we move to develop additional indices for health and nutrition.

### Recommendation

We recommend that the indices included in this review be used as tools for policy and programme stakeholders to measure status, progress and reach of policy and programmes to help guide programme implementation, investment decisions or policy direction. Additionally, there is growing interest in using composite indices to measure population-level coverage of multiple multisectoral nutrition interventions as a means of monitoring the reach of intersectional approaches to impact the health and nutritional status of women and children. This scoping review can be used as a guide for best practices in composite index design and validation.

We propose learnings from this study be used to inform a methodological stepwise framework for constructing composite scores for multisectoral nutrition action. Our forthcoming work in DataDENT^8^ will be producing this framework with validated illustrations from different contexts. The articles identified in this review serve to guide DataDENT’s^8^ development of flexible methods to estimate the layered coverage of multiple interventions across sectors and key populations for the purpose of improving accountability, benchmarking progress and guiding programme and policy design.

Learnings from the current paper, and the forthcoming framework, are useful to health and nutrition policy and programme stakeholders in terms of cataloguing composite health and nutrition indices in the literature and documenting the validation and validation methods of such indices. The indices included in this review can be used as tools for measuring status, progress and reach of policy and programmes to help guide programme implementation, investment decisions or policy direction. This catalogue of indices is also useful to donors who may be provided indices in reporting so that they can better understand the purpose, quality and validity of the indices in use. This work reviews the types, strengths and weaknesses of index formula construction, as well as weighting and validation methods. This is done so that those reviewing, using or designing health and nutrition indices understand the principles, limitations and considerations that underly composite measurement. In addition to policy and programme stakeholders, norm setting groups—such as the WHO—can use such insights to assess the indices in use and make recommendations for best practices when it comes to choice of indices for specific outcomes. The nutrition community more broadly (eg, global summits and initiatives, nutrition collectives and alliances, non-governmental organisations and charitable organisations) working in the space of monitoring progress in nutrition intervention coverage and outcomes can benefit from understanding the evidence and evidence gaps in this space of composite measurement or as a springboard for developing other novel scores for various types of nutrition intervention packages or outcomes. Major global nutrition events and conferences often report on single indicators or composite indicators. However, as this review identified, few indices have been designed with evidence-based standards, reviewed by experts for rationality or statistically validated. Organisers of global events can use the findings of this review as a guide to vetting index quality and reliability when reporting on or designing composite scores for health and nutrition. This is particularly important as there is growing interest in using composite indices to measure population-level coverage of multiple multisectoral nutrition interventions as a means of monitoring the reach of intersectional approaches to impact the health and nutritional status of women and children. Specific areas of consideration are discussed below.

Based on the evidence from this work, we recommend that a hybrid approach of participatory weighting using normative, evidence-based methods and an aggregation using an arithmetic mean be used to develop composite nutrition coverage indices. Additionally, all novel indices must include validation, through at minimum convergent validation and sensitivity analysis, to ensure that the index measures the intended outcome of interest and determine the impact of individual indicators to the overall score. Formula construction methodologies define the approaches used to select indicators and derive weights for individual indicators comprising the index. There are important considerations when choosing formula construction methodologies. Statistical constructions are often less accessible to policy and programme actors due to the complexity and resource requirements of statistical approaches to index development and estimation. The interpretation of statistical approaches also tends to be more convoluted due to poor transparency of the analytical construction and indicator inclusion with this method of construction. This makes indices less flexible for implementation in various contexts where some indicators need to be excluded due to data limitations or programmatic relevance. Normative formula construction is based on normative outcomes, programming or priorities, which may better reflect context. Depending on the type of normative approach, this method of construction can be relatively simple to apply across contexts—in the case of programme-based construction, which relies on number of contacts—or more complex in application—in the case of potential health gains or health expenditure shares. Generally, participatory weighting is simple to interpret and adjust to contexts as needed. However, the challenge posed by participatory weighting is the resource demand of bringing diverse expert perspectives together. There are additional formula construction methods that should be noted but were not identified in the current review of the evidence. Additional statistical approaches include data envelopment analysis, benefit of the doubt approach and unobserved components model.^11^ Other participatory methods include budget allocation process and conjoint analysis.^11^

Aggregation is the process of bringing together individual indicators into a mathematical formula. In the case of composite measures, the analytical formula produces a mean or summary score. Importantly, the arithmetic mean allows for compensation between indicators, meaning that poor performance in one indicator can be compensated by better performance in another indicator. In practice, this means that if a region is prioritising one nutrition intervention at the expense of another, the composite index may mask this inequality in coverage. The geometric mean is typically recommended due to the partial compensation it allows in measuring the mean. Partial compensation means that better performance in one indicator has less of a compensatory effect on poor performance in another indicator. However, in practice, linear aggregation is more common, particularly in the most widely used indices, such as the CCI. Therefore, based on the simplicity of linear aggregation and the success of the CCI, we recommend that linear aggregation methods are used in nutrition index development. Additionally, applying weights to indicators in linear aggregation can help minimise the compensability of the arithmetic mean.

This review identified a paucity in the use of validation methods for metrics. Validation methods provide a basis for generating robust composite measures of health and nutrition that are fit for purpose. There are various types of validation methods that can be used depending on the outcomes of interest, data available and construction methodology. The most common type of validation method is convergent validation, which is primarily implemented through correlation analyses. Depending on the domain the composite score aims to measure, the correlation between the composite score and nutritional outcomes of interest can be used to assess the validity of the derived index. Common correlation covariates include nutrition spending per capita, health outcomes (eg, stunting, under 5 mortality rate and infant mortality), other validated health coverage outcomes (eg, CCI) and development outcomes (eg, HDI). Other validation methods include sensitivity analysis (eg, varying weights for indicators or adding indicators), agreement (eg, agreement of weighting among experts) and cross-validation (eg, the difference between observed and estimated values of the index).

Additional considerations also need to be taken when developing novel indices. A major consideration is the geographical reach and intended utility of the index. For example, in the broader field of public health nutrition many stakeholders want an index that estimates national coverage for cross-country comparisons. This type of index requires a limited number of common, evidence-based sentinel indicators that are common to multiple countries to create an index that can be calculated for multiple regions. Alternatively, if you aimed to advance country-specific coverage estimation that is relevant to country stakeholders, you may want to consider a different mix of contextualised indicators based on policy and programme priorities and interpretability. This more specific and flexible index can then be used to measure country progress based on commitments, which is a growing need as countries expand their use of multisectoral nutrition strategies.

### Strengths and limitations

This review has several limitations. A primary limitation was that only one review author screened and extracted data. Ambiguities were collaboratively addressed in team meetings; however, potential oversights cannot be ruled out. Another limitation was introduced by the language restrictions applied, potentially excluding some relevant studies that were published in languages other than English. A major strength of this scoping review is the systematic search strategy, which compiled a diverse array of studies from multiple disciplines and included grey literature. This review was designed and conducted in accordance with the Joanna Briggs Institute Reviewer Manual^9^ following the PRISMA-ScR verification list.^10^ Supplementary searching, using snowballing to identify other relevant published and grey literature was also used to ensure that the breadth of the literature was captured.

## Conclusion

This scoping review provides an overview of the composite indices currently in use for measuring coverage of multiple health and/or nutrition interventions or health and nutritional status. To our knowledge there are no multisectoral nutrition composite indices currently developed or in use and more than a third of the composite indices currently in use for measuring coverage of multiple health and/or nutrition interventions or health and nutritional status have not been validated. Our review identified a need for additional resources for guiding policy and programme actors to develop fit for purpose composite coverage indices to estimate the layered coverage of multiple nutrition-specific or nutrition-sensitive interventions across sectors and key populations for the purpose of policy design and monitoring. We propose that a methodological framework be developed for stakeholders to guide composite index construction in various contexts.

## Supplementary material

10.1136/bmjopen-2025-111298 online supplemental file 1

10.1136/bmjopen-2025-111298 online supplemental table 1

## Data Availability

Data are available upon reasonable request.
