# Supplementary material for: Measuring multisector nutrition and health intervention coverage using composite coverage analysis methods: a scoping review and methodological guidance
Source: BMJ Open. 2026 Jun 17;16(6):e111298. doi: 10.1136/bmjopen-2025-111298 (PMC13288883; doi:10.1136/bmjopen-2025-111298)
Supplement: online supplemental file 1 [file bmjopen-16-6-s001.pdf]

SCOPING REVIEW PROTOCOL

Title

Measuring multi-sector nutrition and health intervention coverage using composite coverage analysis methods: A scoping review

Authors

Taylor Morrison[1], Nadia Akseer[1], Rebecca A Heidkamp[1], Abdoulaye Maïga[1], Hana Tasic[2]

Author affiliations

1. International Health, Johns Hopkins University Bloomberg School of Public Health, Baltimore, Maryland, USA
2. Modern Scientist Global, St Catharines, Ontario, Canada

Corresponding author: Taylor Morrison (tmorri45@alumni.jh.edu)

Keywords

global health, health equity, sustainable development, nutritional status, health care surveys, health services accessibility, universal health care

Amendments

In the event of protocol amendments, each amendment's date will be reported and accompanied by a description of the change and the rationale.

Funding statement

This work was supported, in whole or in part, by the Gates Foundation [INV-040665]. The conclusions and opinions expressed in this work are those of the author(s) alone and shall not be attributed to the Foundation.

Sponsor

Role of sponsor: The sponsor had no role in the design, data collection, analysis or interpretation of results.

Introduction

Rationale

Many LMICs face challenges in answering questions about who is being reached by multisectoral nutrition programmes. Reach or coverage is a proximal indicator of policy and program implementation that precedes indicators of nutritional status or diet quality outcomes. Collection of coverage measures requires less resources compared to health and nutritional status indicators. Coverage assessments are therefore important for the rapid and iterative processes of programme and policy improvement.

Measures of multi-sector nutrition intervention coverage are needed to monitor the status of nutrition intervention priorities and performance of nutrition-related programmes. There is a need to improve the capacity and readiness to collect and use indicators of multi-sector nutrition intervention coverage in a unified way. Monitoring of multi-sector intervention coverage can help determine and interpret coverage inequalities and inform programme efficiency opportunities within and across sectors. Priority indicators include maternal, infant, and young child nutrition (MIYCN)/maternal supplementation; nutrition-sensitive social protection (NSSP); and large-scale food fortification (LSFF).

Coverage indicators also allow for within- and between-country comparisons and time trend analyses. This can help inform regional priorities, implementation strategies, and global progress. Coverage measures can also provide dissemination tools to advocate for programme and policy change.

Objectives

The aim of this scoping review is to evaluate methodologies for measuring multi-sector nutrition intervention coverage using composite coverage analysis methods. To this end, the proposed scoping review will answer the following questions:

1. Identify composite coverage analysis methods (and co-coverage) used to measure health and nutrition intervention coverage;
2. Describe the general methodologies of composite coverage estimation and their validation approaches; and
3. Evaluate the strengths and weaknesses of various composite coverage analysis methods.

Methods

This scoping review was designed and conducted in accordance with the Joanna Briggs Institute Reviewer Manual [1] following the Preferred Reporting Items for Systematic Reviews and Meta-Analyses Extension for Scoping Reviews (PRISMA-ScR) verification list [2].

Inclusion criteria

Population: All populations will be considered, though maternal and child health nutrition coverage indicators will be prioritized

Concept: Publications describing, assessing, or utilizing composite coverage analysis methods. We will extract indicators included in composite coverage measures as reported in publications. Scoring methods and analytical approaches will be described.

Context: There will be no restrictions by type of setting or context.

Language: We will include articles reported in the English language.

Years considered: 1980 or later

Types of evidence source: Published and grey literature

Publication status: No restrictions

Information sources

Electronic databases, contact with study authors, trial registers, grey literature obtained or published between 1980 and May 18, 2024, will be considered.

Search strategy

PubMed (https://www.ncbi.nlm.nih.gov/pubmed/), EMBASE (https://www.embase.com), Scopus (https://www.scopus.com/), Google Scholar (https://scholar.google.com/), and Global Health (Global Health, 1910 Forward; OvidSP; https://ovidsp.ovid.com/) will be searched. The first 10 pages of results from Google Scholar were extracted. Grey literature will be obtained through a handsearch of organization websites (e.g., Oxford Academic, World Bank, UNICEF, and WHO) and a Google site search of the first five pages of Google results limited to 1980-2024.

The following search strings was used (modified as appropriate for each database) for the provision of composite coverage analysis methods:

Composite and Co-coverage - nutrition and health literature search:

(((composite NEAR/3 index*):ti,ab) OR ((composite NEAR/3 indices):ti,ab) OR ((composite NEAR/3 indicator*):ti,ab) OR ((composite NEAR/3 estimate*):ti,ab) OR ((composite NEAR/3 measure*):ti,ab) OR ((composite NEAR/3 score*):ti,ab) OR ((composite NEAR/3 scoring):ti,ab) OR ((composite NEAR/3 metric*):ti,ab) OR ((composite NEAR/3 framework*):ti,ab) OR ((summary NEAR/3 index):ti,ab) OR ((summary NEAR/3 indices):ti,ab) OR ((summary NEAR/3 indicator):ti,ab) OR ((summary NEAR/3 estimate):ti,ab) OR ((summary NEAR/3 measure):ti,ab) OR ((summary NEAR/3 score):ti,ab) OR ((summary NEAR/3 scoring):ti,ab) OR ((summary NEAR/3 metric):ti,ab) OR ((summary NEAR/3 framework):ti,ab) OR ((aggregate* NEAR/3 index*):ti,ab) OR ((aggregate* NEAR/3 indices):ti,ab) OR ((aggregate* NEAR/3 indicator*):ti,ab) OR ((aggregate* NEAR/3 estimate*):ti,ab) OR ((aggregate* NEAR/3

measure*):ti,ab) OR ((aggregate* NEAR/3 score*):ti,ab) OR ((aggregate* NEAR/3 scoring):ti,ab) OR ((aggregate* NEAR/3 metric*):ti,ab) OR ((aggregate* NEAR/3 framework*):ti,ab) OR ((‘index-based’ NEAR/3 indicator*):ti,ab) OR ((‘index-based’ NEAR/3 estimate*):ti,ab) OR ((‘index-based’ NEAR/3 measure*):ti,ab) OR ((‘index-based’ NEAR/3 score*):ti,ab) OR ((‘index-based’ NEAR/3 scoring):ti,ab) OR ((‘index-based’ NEAR/3 metric*):ti,ab) OR ((‘index-based’ NEAR/3 framework*):ti,ab) OR ((coverage NEAR/3 index*):ti,ab) OR ((coverage NEAR/3 indices):ti,ab) OR ((coverage NEAR/3 indicator*):ti,ab) OR ((coverage NEAR/3 estimate*):ti,ab) OR ((coverage NEAR/3 measure*):ti,ab) OR ((coverage NEAR/3 score*):ti,ab) OR ((coverage NEAR/3 scoring):ti,ab) OR ((coverage NEAR/3 metric*):ti,ab) OR ((coverage NEAR/3 framework*):ti,ab))

AND (method*:ti OR form*:ti,ab OR calculat*:ti,ab OR model*:ti,ab OR technique*:ti,ab OR procedure*:ti,ab OR approach*:ti,ab OR strategy:ti,ab OR strategies:ti,ab OR construct*:ti,ab OR system*:ti,ab OR quantify:ti,ab OR quantified:ti,ab OR quantification:ti,ab OR evaluate*:ti,ab OR develop*:ti,ab OR analysis:ti,ab OR weight*:ti,ab OR slope*:ti,ab)

AND (((nutrition* NEAR/3 intervention*):ti,ab) OR (('nutrition-sensitive' NEAR/3 intervention*):ti,ab) OR ((health NEAR/3 intervention*):ti,ab) OR ((maternal NEAR/3 intervention*):ti,ab) OR ((newborn NEAR/3 intervention*):ti,ab) OR ((child NEAR/3 intervention*):ti,ab) OR ((antenatal NEAR/3 intervention*):ti,ab) OR ((reproductive NEAR/3 intervention*):ti,ab) OR ((social NEAR/3 intervention*):ti,ab) OR ((fortification NEAR/3 intervention*):ti,ab) OR ((nutrition* NEAR/3 coverage*):ti,ab) OR ((health NEAR/3 coverage*):ti,ab) OR ((maternal NEAR/3 coverage*):ti,ab) OR ((newborn NEAR/3 coverage*):ti,ab) OR ((child NEAR/3 coverage*):ti,ab) OR ((vaccin* NEAR/3 coverage*):ti,ab) OR ((antenatal NEAR/3 coverage*):ti,ab) OR ((reproductive NEAR/3 coverage*):ti,ab) OR ((social NEAR/3 coverage*):ti,ab) OR ((fortification NEAR/3 coverage*):ti,ab) OR ((health NEAR/3 indicator*):ti,ab) OR ((nutrition* NEAR/3 indicator*):ti,ab) OR ((coverage NEAR/3 indicator*):ti,ab) OR ((maternal NEAR/3 indicator*):ti,ab) OR ((newborn NEAR/3 indicator*):ti,ab) OR ((child NEAR/3 indicator*):ti,ab) OR ((antenatal NEAR/3 indicator*):ti,ab) OR ((delivery NEAR/3 indicator*):ti,ab) OR ((care NEAR/3 indicator*):ti,ab) OR ((access NEAR/3 indicator*):ti,ab) OR ((utilisation NEAR/3 indicator*):ti,ab) OR ((utilization NEAR/3 indicator*):ti,ab) OR ((vaccin* NEAR/3 indicator*):ti,ab))

Study records

Data management: Literature search results will be uploaded to Covidence. The team will develop and test screening questions and forms for level 1 and 2 assessments based on the inclusion and exclusion criteria. Citation abstracts and full text articles will be uploaded with screening questions to Covidence. Prior to the formal screening process, a calibration exercise will be undertaken to pilot and refine the screening questions.

Selection process: One reviewer will independently screen the titles and abstracts yielded by the search against the inclusion criteria. Full reports will be obtained for titles that appear to meet the

inclusion criteria. One reviewer will then screen full text reports and decide whether they meet the inclusion criteria. We will record reasons for excluding reports.

Data collection process: Standardized forms for data extraction will be piloted and tested. Extracted data and information from each eligible source will be confirmed by the reviewer. Data abstracted will include geographic and demographic information, coverage measure methodology, validation methods, and coverage indicator type. The reviewer will resolve uncertainties by discussion with the co-authors.

Data items

Data item	Definition
Population	Demographic characteristics of the population under study
Concept	Coverage indicator type or outcome of interest
Context	Country, subnational geographical unit, region (urban/rural)
Language	Written language of publication (will only review publications in English)
Date of publication	Date that source was published
Composite assessment method(s)	Type of composite assessment used of methodology developed to measure coverage
Composite assessment formula	Formula used to construct composite score
Indicators used in coverage measure	Indicators and data types used to construct composite score

Outcomes and prioritization

Primary outcomes:

1. Composite coverage estimation approaches
2. Indicators used to measure health and nutrition intervention coverage
3. Scoring methods and analytical approaches of composite measures

Secondary outcomes:

4. Feasibility, strengths, and limitations of composite coverage analysis methods
5. Validation/comparison assessment methods for composite coverage measures

Data synthesis

Outputs will include findings as publications and guidance on how to develop and apply co-coverage and composite coverage indicator methods. Composite coverage and validation methodologies will be applied to previously collected data from Ethiopia.

References

[1] Aromataris E, Lockwood C, Porritt K, et al., editors. JBI Manual for Evidence Synthesis. JBI; 2024. https://doi.org/10.46658/JBIMES-24-01.

[2] PRISMA Extension for Scoping Reviews (PRISMA-ScR): Checklist and Explanation. Ann Intern Med 2018;169:467–73. https://doi.org/10.7326/M18-0850.
