# Supplementary material for: Measuring multisector nutrition and health intervention coverage using composite coverage analysis methods: a scoping review and methodological guidance
Source: BMJ Open. 2026 Jun 17;16(6):e111298. doi: 10.1136/bmjopen-2025-111298 (PMC13288883; doi:10.1136/bmjopen-2025-111298)
Supplement: online supplemental table 1 [file bmjopen-16-6-s002.pdf]

Supplementary Table 1. Composite indices discovered (n=56)

Index (no. of times cited)	Domain of measurement	Geographical application	Population measured	Formula construction methodology	Formula aggregation	Citations with validation
BC Adolescent Health And Wellness Index (BCAHWI) (n=1) [1]	Health Services	British Columbia, Canada	Adolescents	Participatory (expert opinion, Delphi)	Weighted arithmetic mean	1
Child Health Index (CHI) (n=1) [2]	Universal Healthcare	Sweden	Children 0-17 years	Statistical (Factor analysis)	Weighted arithmetic mean	1
Chinese UHC Index (Index Of Accessibility, Index Of Affordability) (n=1) [3]	Universal Healthcare	China	All people	Normative (equal weighting)	Geometric mean	1
Composite Coverage Index (CCI) (n=45) [4–48]	RMNCH	Global	Mothers and children	Normative (interventions, number of contacts)	Weighted arithmetic mean	9
CCI, modified (Ethiopia) (n=1) [49]	RMNCH	Ethiopia	Mothers and children	Normative (interventions, number of contacts)	Weighted arithmetic mean	0
CCI, modified (India) (n=1) [50]	RMNCH	India	Mothers and children	Normative (interventions, number of contacts)	Weighted arithmetic mean	0
CCI, Modified ASHA-Centric CCI (MACCI) (n=2) [51,52]	RMNCH	India	Mothers and children	Normative (equal weighting)	Weighted arithmetic mean	0
Composite Health Risk Factor Index (HRFI) (composed of the following subindices: Nutrition Index (NUTI), Environmental Risk Factors Index (ERFI), and Noncommunicable Disease Index (NCDI)) (n=1) [53]	Universal Health Care; Health risk factors; Nutrition	Global	All people	Statistical (PCA)	Weighted arithmetic mean	1
Composite Index Of Health (CIH) (n=1) [54]	Universal Healthcare	India	All people	Normative (equal weighting), Statistical (PCA)	Geometric mean	1
Composite Index Of Health Inequity (IIS) (n=1) [55]	Health Services	Colombia	All people	Statistical (PCA)	Weighted arithmetic mean	1
Composite NCD Management Index (n=1) [56]	Universal Healthcare	China	All people	Normative (WHO guideline)	Random-effects meta-analysis	1
Composite NCD Prevention Index (n=1) [56]	Universal Healthcare	China	All people	Normative (WHO guideline)	Random-effects meta-analysis	1
Composite NCD Treatment Index (n=1) [56]	Universal Healthcare	China	All people	Normative (WHO guideline)	Random-effects meta-analysis	1
Composite Prevention Index (n=5) [21,30,32,44,57]	Universal Healthcare	Global	All people	Normative (WHO guideline)	Random-effects meta-analysis	1
Composite Treatment Index (n=5) [21,30,32,44,57]	Universal Healthcare	Global	All people	Normative (WHO guideline)	Random-effects meta-analysis	1
Composite UHC Index (n=1) [58]	Universal Healthcare	Asia and Africa	All people	Normative (based on geometric measure	Arithmetic mean	1

Index (no. of times cited)	Domain of measurement	Geographical application	Population measured	Formula construction methodology	Formula aggregation	Citations with validation
				of the three dimensions of UHC)		
Composite UHC Index (CUHCI) (n=1) [59]	Universal Healthcare	India	All people	Statistical (PCA)	Geometric mean	1
Comprehensive Continuum of Care for Maternal Health Index (C3MH Index) (n=1) [60]	RMNCH	South Africa	Mothers	Normative (interventions, data availability)	Geometric mean	1
Coverage Gap Index (CGI) (n=8) [61–68]	RMNCH	Global	Mothers and children	Normative (interventions, number of contacts)	Weighted arithmetic mean	4
Global Hunger Index (GHI) (n=4) [69–72]	Nutrition	Global	All people	Statistical (PCA)	Arithmetic mean	1
Global Hunger Index (GHI) (formerly, the International Nutrition Index (NI)) (n=1) [73]	Nutrition	Global	All people	Statistical (factor analysis)	Weighted arithmetic mean	1
Global Hunger Index (GHI), revised (n=1) [69]	Nutrition	Global	All people	Statistical (PCA)	Weighted arithmetic mean	1
Global Nutritional Index (GNI) (n=1) [74]	Nutrition	LMICs	All people	Normative (equal weighting)	Arithmetic mean	0
Global One Health Index-Food Security (GOHI-FS) (n=1) [75]	Nutrition	Global	All people	Participatory (expert opinion, FAHP)	Weighted arithmetic mean	0
Health Opportunity Index (HOI) (n=1) [76]	Health Services	United States	All people	Statistical (PCA)	Weighted arithmetic mean	1
Health Service Coverage Index (n=1) [77]	Health Services	Global	All people	Statistical (PCA)	Weighted arithmetic mean	1
Hidden Hunger Index (n=1) [78]	Nutrition	Global	Children under five	Normative (contribution to hidden hunger and data availability)	Arithmetic mean	1
Hybrid Mixed Index (Hybrid-MI) (n=1) [79]	RMNCH	LMICs	Mothers and children	Statistical (Hybrid double reference point (DRP) and Pena's distance P2 (DP2) methodology)	Weighted arithmetic mean	0
Hybrid Strong Index (Hybrid-SI) (n=1) [79]	RMNCH	LMICs	Mothers and children	Statistical (Hybrid double reference point (DRP) and Pena's distance P2 (DP2) methodology)	Weighted arithmetic mean	0
Hybrid Weak Index (Hybrid-WI) (n=1) [79]	RMNCH	LMICs	Mothers and children	Statistical (Hybrid double reference point (DRP) and Pena's distance P2 (DP2) methodology)	Weighted arithmetic mean	0
Input-Adjusted Coverage (n=1) [80]	Nutrition	Bangladesh	Mothers and children	Normative (equal weighting)	Weighted arithmetic mean	0
Iran Social Health Index (n=1) [81]	Universal Healthcare	Iran	All people	Participatory (expert opinion)	Weighted arithmetic mean	1
Korean Community Health Determinants Index (K-CHDI) (n=1) [82]	Health Services	Korea	All people	Participatory (expert opinion, AHP)	Weighted arithmetic mean	1

Index (no. of times cited)	Domain of measurement	Geographical application	Population measured	Formula construction methodology	Formula aggregation	Citations with validation
Maternal Health Service Coverage Index (MHSI) (n=1) [83]	RMNCH	India	Mothers	Normative (equal weighting)	Geometric mean	0
MDG Coverage Score (n=1) [84]	Universal Healthcare	Tanzania	All people	Normative (interventions, data availability)	Weighted arithmetic mean	0
MDG Index (n=1) [85]	Universal Healthcare	Global	All people	Participatory (expert opinion)	Geometric mean	1
Non-MDG Index (n=1) [85]	Universal Healthcare	Global	All people	Participatory (expert opinion)	Geometric mean	1
MNCH Continuum Of Care Coverage (n=1) [86]	RMNCH	China	Mothers and children	Normative (interventions, number of contacts)	Weighted arithmetic mean	1
Net State of Nutrition Index (NeSNI) (n=2) [87,88]	Nutrition	Global	Mothers and children	Normative (equal weighting)	Standardized and summed	1
Net State Of Nutrition Index (NeSNI)-Triple Burden (N=1) [87]	Nutrition	Global	Mothers and children	Normative (equal weighting)	Standardized and summed	0
NITI Aayog Health Index (n=1) [89]	Universal Healthcare	India	All people	Participatory (expert opinion)	Weighted arithmetic mean	0
Nutritional Index (n=1) [90]	Nutrition	India	Children under 5 years	Normative (weight based on impact on nutritional status)	Weighted arithmetic mean	0
Overall Health-Related SDG Index (n=1) [85]	Universal Healthcare	Global	All people	Participatory (expert opinion)	Geometric mean	1
Overall Intervention Coverage (n=2) [91,92]	RMNCH	Uganda, Nigeria	Mothers and children	Normative (equal weighting)	Arithmetic mean	0
Overall Measure of Composite Coverage (n=1) [93]	RMNCH	Zambia	Mothers and children	Normative (equal weighting)	Arithmetic mean	0
Public Health Development Index (PHDI) (n=1) [94]	Health Services	Indonesia	All people	Participatory (expert opinion)	Arithmetic mean	0
RMNCH Health Service Coverage and sub-indices (Prevention and Treatment Sub-Indices) (n=1) [95]	RMNCH	Vietnam	Mothers and children	Normative (WHO guideline)	Geometric mean; Random-effects meta-analysis (sub-indices)	1
UHC Assessment Index (n=1) [96]	Universal Healthcare	Bhutan	All people	Normative (interventions, weighted based on the contribution to the achievement of the policy goals of UHC)	Weighted arithmetic mean	0
UHC Effective Coverage Index (n=11) [97–107]	Universal Healthcare	Global	All people	Normative (weight based on health gains)	Weighted arithmetic mean	2
UHC Index (SDG Indicator 3.8.1) (n=1) [108]	Universal Healthcare	Global	All people	Participatory (expert opinion, preference-weighted by relative importance)	Arithmetic mean	0
UHC Index and associated sub-indices (Service Coverage Index— reproductive,	Universal Healthcare	Global	All people	Normative (interventions, weighted based on	Weighted geometric mean	4

Index (no. of times cited)	Domain of measurement	Geographical application	Population measured	Formula construction methodology	Formula aggregation	Citations with validation
maternal, newborn and child health (RMNCH); infectious diseases (ID); non-communicable diseases (NCD); and service capacity and access (SCA)—and Financial Protection Index— catastrophic out-of-pocket (OOP) health expenditure (CATA) and impoverishing OOP healthcare expenditure (IMPOV)) (n=10) [109–118]				health expenditure shares)		
Modified UHC index (Pakistan) (n=1) [119]	Universal Healthcare	Pakistan	All people	Normative (equal weighting)	Geometric mean	1
Modified hybrid of the UHC index and UHC SCI indices and sub-indices (Reproductive, maternal, neonatal and child health subindex; Infectious disease subindex; NCD sub-index; Service capacity and access subindex) (China) (n=1) [120]	Universal Healthcare	China	All people	Normative (equal weighting)	Geometric mean	1
UHC Service Coverage Index (UHC SCI) and associated sub-indices (reproductive, maternal, neonatal and child health subindex; infectious disease subindex; NCD sub-index; and service capacity and access subindex) (n=33) [101,121–152]	Universal Healthcare; RMNCH; Health services	Global	All people; Mothers and children; all people 15 years or older; all people	Normative (equal weighting)	Geometric mean	3
Modified UHC SCI and sub-indices (Reproductive, maternal, neonatal and child health subindex; Infectious disease subindex; NCD sub-index; Service capacity and access) (India) (n=1) [153]	Universal Healthcare	India	All people	Normative (equal weighting)	Geometric mean	1
Vulnerability Index (n=1) [154]	Universal Healthcare	India	All people	Normative (equal weighting)	Arithmetic mean	0

References

[1] Martin G, Keller CP, Foster LT. Constructing a composite adolescent health and wellness index for British Columbia, Canada using a spatial multi-criteria analysis approach. Child Indicators Research 2012;5:215–34. https://doi.org/10.1007/s12187-011-9129-z.

[2] Köhler L, Eriksson B. A Child Health Index for Sweden’s 290 Municipalities: A System of Indicators and Indices for Monitoring Children’s Health on the Local Level. Child Indic Res 2018;11:1889–906. https://doi.org/10.1007/s12187-017-9515-2.

[3] Liu X, Wang Z, Zhang H, et al. Measuring and evaluating progress towards Universal Health Coverage in China. Journal of Global Health 2021;11:8005. https://doi.org/10.7189/jogh.11.08005.

[4] Akseer N, Bhatti Z, Rizvi A, et al. Coverage and inequalities in maternal and child health interventions in Afghanistan. BMC Public Health 2016;16 Suppl 2:797. https://doi.org/10.1186/s12889-016-3406-1.

[5] Akseer N, Rizvi A, Bhatti Z, et al. Association of Exposure to Civil Conflict with Maternal Resilience and Maternal and Child Health and Health System Performance in Afghanistan. JAMA Network Open 2019;2. https://doi.org/10.1001/jamanetworkopen.2019.14819.

[6] Barros AJD, Wehrmeister FC, Ferreira LZ, et al. Are the poorest poor being left behind? Estimating global inequalities in reproductive, maternal, newborn and child health. BMJ Glob Health 2020;5:e002229. https://doi.org/10.1136/bmjgh-2019-002229.

[7] Barros AJ, Ronsmans C, Axelson H, et al. Equity in maternal, newborn, and child health interventions in Countdown to 2015: A retrospective review of survey data from 54 countries. The Lancet 2012;379:1225–33. https://doi.org/10.1016/S0140-6736(12)60113-5.

[8] Barros AJ, Victora CG. Measuring coverage in MNCH: determining and interpreting inequalities in coverage of maternal, newborn, and child health interventions. PLoS Medicine 2013;10:e1001390.

[9] Baye K, Laillou A, Chitweke S. Socio-economic inequalities in child stunting reduction in sub-Saharan Africa. Nutrients 2020;12. https://doi.org/10.3390/nu12010253.

[10] Blanchard AK, Jacobs C, Musukuma M, et al. Going deeper with health equity measurement: how much more can surveys reveal about inequalities in health intervention coverage and mortality in Zambia? International Journal for Equity in Health 2023;22. https://doi.org/10.1186/s12939-023-01901-x.

[11] Cisse D, Toure AA, Diallo A, et al. Evaluation of maternal and child care continuum in guinea: a secondary analysis of two demographic and health surveys using the composite coverage index (CCI). BMC Pregnancy and Childbirth 2023;23. https://doi.org/10.1186/s12884-023-05718-y.

[12] Colomé-Hidalgo M, Campos JD, Miguel ÁG de. Exploring wealth-related inequalities in maternal and child health coverage in Latin America and the Caribbean. BMC Public Health 2021;21:115. https://doi.org/10.1186/s12889-020-10127-3.

[13] Corsi DJ, Subramanian SV. Association between coverage of maternal and child health interventions, and under-5 mortality: a repeated cross-sectional analysis of 35 sub-Saharan African countries. Global Health Action 2014;7:24765. https://doi.org/10.3402/gha.v7.24765.

[14] Ewerling F, Wehrmeister FC, Victora CG, et al. Is women’s empowerment associated with coverage of RMNCH interventions in low- and middle-income countries? An analysis

using a survey-based empowerment indicator, the SWPER. Journal of Global Health 2021;11:4015. https://doi.org/10.7189/jogh.11.04015.
[15] Faye CM, Wehrmeister FC, Melesse DY, et al. Large and persistent subnational inequalities in reproductive, maternal, newborn and child health intervention coverage in sub-Saharan Africa. BMJ Global Health 2020;5. https://doi.org/10.1136/bmjgh-2019-002232.
[16] Ferreira LZ, Utazi CE, Huicho L, et al. Geographic inequalities in health intervention coverage - mapping the composite coverage index in Peru using geospatial modelling. BMC Public Health 2022;22:2104. https://doi.org/10.1186/s12889-022-14371-7.
[17] Gandhi S, Maharatha TM, Dash U, et al. Level of inequality and the role of governance indicators in the coverage of reproductive maternal and child healthcare services: Findings from India. PLoS ONE 2021;16. https://doi.org/10.1371/journal.pone.0258244.
[18] Garchitorena A, Miller AC, Cordier LF, et al. Early changes in intervention coverage and mortality rates following the implementation of an integrated health system intervention in Madagascar. BMJ Glob Health 2018;3:e000762. https://doi.org/10.1136/bmjgh-2018-000762.
[19] Gebremedhin AF, Dawson A, Hayen A. Determinants of continuum of care for maternal, newborn, and child health services in Ethiopia: Analysis of the modified composite coverage index using a quantile regression approach. PLoS ONE 2023;18. https://doi.org/10.1371/journal.pone.0280629.
[20] Gebreselassie T, Wang WenJuan, Sougane A, et al. Inequalities in the coverage of reproductive, maternal, newborn, and child health interventions in Mali: further analysis of the Mali Demographic and Health Surveys 2006-2018. DHS Further Analysis Report 2020:xi-pp.
[21] Han SM, Rahman MM, Rahman MS, et al. Progress towards universal health coverage in Myanmar: a national and subnational assessment. Lancet Glob Health 2018;6:e989–97. https://doi.org/10.1016/S2214-109X(18)30318-8.
[22] Hasan MM, Magalhaes RJS, Ahmed S, et al. Meeting the global target in reproductive, maternal, newborn, and child health care services in low- and middle-income countries. Global Health: Science and Practice 2020;8:654–65. https://doi.org/10.9745/GHSP-D-20-00097.
[23] Huicho L, Segura ER, Huayanay-Espinoza CA, et al. Child health and nutrition in Peru within an antipoverty political agenda: A Countdown to 2015 country case study. The Lancet Global Health 2016;4:e414–26. https://doi.org/10.1016/S2214-109X(16)00085-1.
[24] Kachoria AG, Mubarak MY, Singh AK, et al. The association of religion with maternal and child health outcomes in South Asian countries. PLoS One 2022;17:e0271165. https://doi.org/10.1371/journal.pone.0271165.
[25] Keats EC, Akseer N, Bhatti Z, et al. Assessment of Inequalities in Coverage of Essential Reproductive, Maternal, Newborn, Child, and Adolescent Health Interventions in Kenya. JAMA Netw Open 2018;1:e185152. https://doi.org/10.1001/jamanetworkopen.2018.5152.
[26] Mujica OJ, Sanhueza A, Carvajal-Velez L, et al. Recent trends in maternal and child health inequalities in Latin America and the Caribbean: analysis of repeated national surveys. International Journal for Equity in Health 2023;22. https://doi.org/10.1186/s12939-023-01932-4.
[27] Oh J, Moon J, Choi JW, et al. Factors associated with the continuum of care for maternal, newborn and child health in The Gambia: a cross-sectional study using Demographic and

Health Survey 2013. BMJ Open 2020;10:e036516-. https://doi.org/10.1136/bmjopen-2019-036516.
[28] Pandey AR, Ojha B, Shrestha N, et al. Progress in Reducing Inequalities in Reproductive, Maternal, Newborn and Child Health Services in Nepal. Journal of Nepal Health Research Council 2021;19:140–7. https://doi.org/10.33314/jnhrc.v19i1.3375.
[29] Parvin N, Rahman M, Islam MJ, et al. Socioeconomic inequalities in the continuum of care across women's reproductive life cycle in Bangladesh. Scientific Reports 2022;12:15618. https://doi.org/10.1038/s41598-022-19888-w.
[30] Rahman MM, Karan A, Rahman MS, et al. Progress toward universal health coverage: A comparative analysis in 5 South Asian countries. JAMA Internal Medicine 2017;177:1297–305. https://doi.org/10.1001/jamainternmed.2017.3133.
[31] Rahman MM, Rouyard T, Khan ST, et al. Reproductive, maternal, newborn, and child health intervention coverage in 70 low-income and middle-income countries, 2000-30: trends, projections, and inequities. Lancet Global Health 2023;11:e1531–43. https://doi.org/10.1016/S2214-109X(23)00358-3.
[32] Rahman MS, Rahman MM, Gilmour S, et al. Trends in, and projections of, indicators of universal health coverage in Bangladesh, 1995–2030: a Bayesian analysis of population-based household data. The Lancet Global Health 2018;6:e84–94. https://doi.org/10.1016/S2214-109X(17)30413-8.
[33] Restrepo-Méndez MC, Barros AJ, Requejo J, et al. Progress in reducing inequalities in reproductive, maternal, newborn,' and child health in Latin America and the Caribbean: an unfinished agenda. Revista Panamericana de Salud Publica = Pan American Journal of Public Health 2015;38:9–16.
[34] Singh PK, Rai RK, Kumar C. Equity in maternal, newborn, and child health care coverage in India. Global Health Action 2013;6:22217. https://doi.org/10.3402/gha.v6i0.22217.
[35] Thapa J, Budhathoki SS, Gurung R, et al. Equity and Coverage in the Continuum of Reproductive, Maternal, Newborn and Child Health Services in Nepal-Projecting the Estimates on Death Averted Using the LiST Tool. Maternal and Child Health Journal 2020;24:22–30. https://doi.org/10.1007/s10995-019-02828-y.
[36] Thomson DR, Amoroso C, Atwood S, et al. Impact of a health system strengthening intervention on maternal and child health outputs and outcomes in rural Rwanda 2005-2010. BMJ Glob Health 2018;3:e000674. https://doi.org/10.1136/bmjgh-2017-000674.
[37] Victora CG, Barros AJD, Axelson H, et al. How changes in coverage affect equity in maternal and child health interventions in 35 Countdown to 2015 countries: An analysis of national surveys. The Lancet 2012;380:1149–56. https://doi.org/10.1016/S0140-6736(12)61427-5.
[38] Victora CG, Barros AJD, França GVA, et al. The contribution of poor and rural populations to national trends in reproductive, maternal, newborn, and child health coverage: analyses of cross-sectional surveys from 64 countries. The Lancet Global Health 2017;5:e402–7. https://doi.org/10.1016/S2214-109X(17)30077-3.
[39] Vidaletti LP, Cata-Preta BO, Phillips DE, et al. Time trends in ethnic inequalities in child health and nutrition: analysis of 59 low and middle-income countries. International Journal for Equity in Health 2023;22. https://doi.org/10.1186/s12939-023-01888-5.
[40] Wehrmeister FC, Silva ICM da, Barros AJD, et al. Is governance, gross domestic product, inequality, population size or country surface area associated with coverage and equity of

health interventions? Ecological analyses of cross-sectional surveys from 80 countries. BMJ Global Health 2017;2:e000437. https://doi.org/10.1136/bmjgh-2017-000437.
[41] Wehrmeister FC, Barros AJD, Hosseinpoor AR, et al. Measuring universal health coverage in reproductive, maternal, newborn and child health: an update of the composite coverage index. PLoS ONE 2020;15. https://doi.org/10.1371/journal.pone.0232350.
[42] Wehrmeister FC, Fayé CM, Silva ICM da, et al. Wealth-related inequalities in the coverage of reproductive, maternal, newborn and child health interventions in 36 countries in the African region. Bulletin of the World Health Organization 2020;98:394–405. https://doi.org/10.2471/BLT.19.249078.
[43] Wehrmeister FC, Restrepo-Mendez M-C, Franca GVA, et al. Summary indices for monitoring universal coverage in maternal and child health care. Bulletin of the World Health Organization 2016;94:903–12. https://doi.org/10.2471/BLT.16.173138.
[44] Zhang C, Rahman MdS, Rahman MdM, et al. Trends and projections of universal health coverage indicators in Ghana, 1995-2030: A national and subnational study. PLoS ONE 2019;14:e0209126. https://doi.org/10.1371/journal.pone.0209126.
[45] Amboko B, Novignon J, Muthuri RNDK, et al. Level and determinants of district primary healthcare system technical efficiency in Ghana: two-stage stochastic frontier analysis. BMJ Glob Health 2026;11:e018847. https://doi.org/10.1136/bmjgh-2024-018847.
[46] Groteclaes T, Ahmed S, Blumenberg C, et al. Equity of maternal and child health services in Afghanistan: a spatiotemporal analysis of national survey datasets. BMJ Glob Health 2025;10:e018577. https://doi.org/10.1136/bmjgh-2024-018577.
[47] Mendez-Lopez A, Mak R. Wealth-based inequalities in the coverage of reproductive, maternal, newborn and child health interventions in 19 countries of the Asia-Pacific region, 1994–2022. Public Health 2025;248:105941. https://doi.org/10.1016/j.puhe.2025.105941.
[48] Rahman MdM, Ii M, Lohan M, et al. Progress towards universal health coverage in South Asia, 2000–2030: an examination of the twin elements of primary healthcare provision and financial protection. BMJ Glob Health 2025;10:e020052. https://doi.org/10.1136/bmjgh-2025-020052.
[49] Abadula KH, Worku AG, Debelew GT, et al. Impact of Health Information System Interventions on Maternal Health Service Utilization in Oromia and Gambella Regions, Ethiopia: A Comparative Cross-Sectional Study. Digit Health 2026;12:20552076261417851. https://doi.org/10.1177/20552076261417851.
[50] S. R, Neethi Mohan V, Vaidyanathan G, et al. Wealth and education-related inequalities in the utilisation of reproductive, maternal, newborn, and child health interventions within scheduled tribes in India: an analysis of Odisha and Jharkhand. BMC Public Health 2024;24:1605. https://doi.org/10.1186/s12889-024-18857-4.
[51] Modi D, Dholakia N, Gopalan R, et al. mHealth intervention “ImTeCHO” to improve delivery of maternal, neonatal, and child care services-A cluster-randomized trial in tribal areas of Gujarat, India. PLoS Med 2019;16:e1002939. https://doi.org/10.1371/journal.pmed.1002939.
[52] Modi D, Desai S, Dave K, et al. Cluster randomized trial of a mHealth intervention “ImTeCHO” to improve delivery of proven maternal, neonatal, and child care interventions through community-based Accredited Social Health Activists (ASHAs) by enhancing their motivation and strengthening. Trials 2017;18. https://doi.org/10.1186/s13063-017-1998-0.

[53] Junaid K, Kiran T, Gupta M, et al. Trends and patterns of global health risk factors (2015–2019): a composite index approach across 100 countries stratified by human development index groups. J Health Popul Nutr 2025;44:357. https://doi.org/10.1186/s41043-025-00945-9.
[54] Panda BK, Mohanty SK. PROGRESS AND PROSPECTS OF HEALTH-RELATED SUSTAINABLE DEVELOPMENT GOALS IN INDIA. Journal of Biosocial Science 2019;51:335–52. https://doi.org/10.1017/S0021932018000202.
[55] Rincón CJ, Pinzón CE, Villada AC, et al. Composite index of health inequity for a middle income country. Revista de Salud Publica 2017;19:249–57.
[56] Zhou Y, Wu Q, Li C, et al. Inequalities in non-communicable disease management in China and progress toward universal health coverage: an analysis of nationwide household survey data from 2004 to 2018. The Lancet Regional Health - Western Pacific 2024;44:100989. https://doi.org/10.1016/j.lanwpc.2023.100989.
[57] Li Y, Zhang C, Zhan P, et al. Trends and projections of universal health coverage indicators in China, 1993–2030: An analysis of data from four nationwide household surveys. The Lancet Regional Health - Western Pacific 2023;31. https://doi.org/10.1016/j.lanwpc.2022.100646.
[58] Khan JAM, Ahmed S, Chen T, et al. A Transparent Universal Health Coverage Index with Decomposition by Socioeconomic Groups: Application in Asian and African Settings. Applied Health Economics and Health Policy 2019. https://doi.org/10.1007/s40258-019-00464-9.
[59] Prinja S, Gupta R, Bahuguna P, et al. A composite indicator to measure universal health care coverage in India: way forward for post-2015 health system performance monitoring framework. Health Policy and Planning 2017;32:43–56.
[60] Mothupi MC, Man JD, Tabana1 H, et al. Development and testing of a composite index to monitor the continuum of maternal health service delivery at provincial and district level in South Africa. PLoS ONE 2021;16. https://doi.org/10.1371/journal.pone.0252182.
[61] Boerma JT, Bryce J, Kinfu Y, et al. Mind the gap: equity and trends in coverage of maternal, newborn, and child health services in 54 Countdown countries. The Lancet 2008;371:1259–67. https://doi.org/10.1016/S0140-6736(08)60560-7.
[62] Awasthi A, Pandey CM, Chauhan RK, et al. Disparity in maternal, newborn and child health services in high focus states in India: A district-level cross-sectional analysis. BMJ Open 2016;6. https://doi.org/10.1136/bmjopen-2015-009885.
[63] Hosseinpoor AR, Victora CG, Bergen N, et al. Towards universal health coverage: The role of within-country wealth-related inequality in 28 countries in sub-Saharan Africa. Bulletin of the World Health Organization 2011;89:881–90. https://doi.org/10.2471/BLT.11.087536.
[64] Kumar C, Singh PK, Rai RK. Coverage gap in maternal and child health services in India: Assessing trends and regional deprivation during 1992-2006. Journal of Public Health (United Kingdom) 2013;35:598–606. https://doi.org/10.1093/pubmed/fds108.
[65] Panda BK, Kumar G, Awasthi A. District level inequality in reproductive, maternal, neonatal and child health coverage in India. BMC Public Health 2020;20:58. https://doi.org/10.1186/s12889-020-8151-9.
[66] Rai RK, Kumar C, Singh PK. District level coverage gap in Maternal, Newborn and Child Health care services in India. Journal of Epidemiology and Global Health 2012;2:221–4. https://doi.org/10.1016/j.jegh.2012.12.004.

[67] Taqi M, Sarkar S, Khan MMA. Analyzing the disparities in the coverage of maternal and child health services: A district-level cross-sectional analysis of Jammu and Kashmir. Indian Journal of Public Health 2020;64:130–4. https://doi.org/10.4103/ijph.IJPH_103_19.

[68] Boerma T, AbouZahr C, Evans D, et al. Monitoring Intervention Coverage in the Context of Universal Health Coverage. PLoS Med 2014;11:e1001728. https://doi.org/10.1371/journal.pmed.1001728.

[69] Wiesmann D, Biesalski HK, Von Grebmer K, et al. Methodological Review and Revision of the Global Hunger Index. SSRN Journal 2015. https://doi.org/10.2139/ssrn.2673491.

[70] von Grebmer K, Headey D, Béné C, et al. 2013 Global Hunger Index The challenge of hunger: Building resilience to achieve food and nutrition security. 0 ed. Washington, DC: International Food Policy Research Institute; 2013. https://doi.org/10.2499/9780896299511.

[71] Wiesmann D. A global hunger index: measurement concept, ranking of countries, and trends. Washington, D.C.: International Food Policy Research Institute (IFPRI); 2006.

[72] Wiesmann D, Weingärtner L, Schöninger I. Global Hunger Index: The challenge of hunger: Facts, determinants, and trends. Bonn, Washington, D.C.: Welthungerhilfe, International Food Policy Research Institute (IFPRI); 2006.

[73] Wiesmann D, Von Braun J, Feldbrügge T. An International Nutrition Index – Successes and Failures in Addressing Hunger and Malnutrition. SSRN Journal 2000. https://doi.org/10.2139/ssrn.3336590.

[74] Rosenbloom JI, Kaluski DN, Berry EM. A global nutritional index. Food Nutr Bull 2008;29:266–77. https://doi.org/10.1177/156482650802900403.

[75] Gu SY, Chen FM, Zhang CS, et al. Assessing food security performance from the One Health concept: an evaluation tool based on the Global One Health Index. Infect Dis Poverty 2023;12:88. https://doi.org/10.1186/s40249-023-01135-7.

[76] Ogojiaku CN, Allen J, Anson-Dwamena R, et al. The health opportunity index: Understanding the input to disparate health outcomes in vulnerable and high-risk census tracts. International Journal of Environmental Research and Public Health 2020;17:5767.

[77] Leegwater A, Wong W, Avila C. A concise, health service coverage index for monitoring progress towards universal health coverage. BMC Health Services Research 2015;15:230. https://doi.org/10.1186/s12913-015-0859-3.

[78] Muthayya S, Rah JH, Sugimoto JD, et al. The Global Hidden Hunger Indices and Maps: An Advocacy Tool for Action. PLoS ONE 2013;8:e67860. https://doi.org/10.1371/journal.pone.0067860.

[79] Luque M, Pérez-Moreno S, Robles JA, et al. Measuring Child and Maternal Health in Developing Countries: A Proposal of New Hybrid MDG Composite Indices. Applied Research in Quality of Life 2017;12:737–58. https://doi.org/10.1007/s11482-016-9487-2.

[80] Nguyen PH, Khuong LQ, Pramanik P, et al. Effective coverage of nutrition interventions across the continuum of care in Bangladesh: Insights from nationwide cross-sectional household and health facility surveys. BMJ Open 2021;11. https://doi.org/10.1136/bmjopen-2020-040109.

[81] Zamankhani F, Abachizadeh K, Omidnia S, et al. Composite social health index: Development and assessment in provinces of Iran. Med J Islam Repub Iran 2019;33:78. https://doi.org/10.34171/mjiri.33.78.

[82] Go DS, Kim YE, Yoon SJ. Development of the Korean Community Health Determinants Index (K-CHDI). PLoS One 2020;15:e0240304. https://doi.org/10.1371/journal.pone.0240304.
[83] Kiran T, Junaid KP, Rajagopal V, et al. Measurement and mapping of maternal health service coverage through a novel composite index: a sub-national level analysis in India. BMC Pregnancy and Childbirth 2022;22. https://doi.org/10.1186/s12884-022-05080-5.
[84] Kumalija CJ, Perera S, Masanja H, et al. Regional differences in intervention coverage and health system strength in Tanzania. PLoS ONE 2015;10. https://doi.org/10.1371/journal.pone.0142066.
[85] Lim SS, Allen K, Dandona L, et al. Measuring the health-related Sustainable Development Goals in 188 countries: a baseline analysis from the Global Burden of Disease Study 2015. The Lancet 2016;388:1813–50. https://doi.org/10.1016/S0140-6736(16)31467-2.
[86] Wang Y, Liao R, Feng XL. Equity in Essential Maternal, Newborn, and Child Health Interventions in Northeastern China, 2008 to 2018. Front Public Health 2020;8:212. https://doi.org/10.3389/fpubh.2020.00212.
[87] Luo H, Zyba SJ, Webb P. Measuring malnutrition in all its forms: An update of the net state of nutrition index to track the global burden of malnutrition at country level. Global Food Security 2020;26. https://doi.org/10.1016/j.gfs.2020.100453.
[88] Webb P, Luo H, Gentilini U. Measuring multiple facets of malnutrition simultaneously: the missing link in setting nutrition targets and policymaking. Food Security 2015;7:479–92. https://doi.org/10.1007/s12571-015-0450-0.
[89] Kamath R, Lakshmi V, Brand H. Health index scores and health insurance coverage across India: A state level spatiotemporal analysis. Clinical Epidemiology and Global Health 2022;18. https://doi.org/10.1016/j.cegh.2022.101185.
[90] Agarwal N, Chaudhary N, Pathak PK, et al. Composite Indexing for Nutritional Status Evaluation: A Snapshot of Malnutrition across India. Indian J Community Med 2020;45:343–7. https://doi.org/10.4103/ijcm.IJCM_387_19.
[91] Roberts DA, Ng M, Ikilezi G, et al. Benchmarking health system performance across regions in Uganda: A systematic analysis of levels and trends in key maternal and child health interventions, 1990-2011. BMC Medicine 2015;13. https://doi.org/10.1186/s12916-015-0518-x.
[92] Wollum A, Burstein R, Fullman N, et al. Benchmarking health system performance across states in Nigeria: a systematic analysis of levels and trends in key maternal and child health interventions and outcomes, 2000-2013. BMC Med 2015;13:208. https://doi.org/10.1186/s12916-015-0438-9.
[93] Colson KE, Dwyer-Lindgren L, Achoki T, et al. Benchmarking health system performance across districts in Zambia: A systematic analysis of levels and trends in key maternal and child health interventions from 1990 to 2010. BMC Medicine 2015;13. https://doi.org/10.1186/s12916-015-0308-5.
[94] Suparmi, Kusumawardani N, Nambiar D, et al. Subnational regional inequality in the Public Health Development Index in Indonesia. Special Issue: Monitoring Health Inequality in Indonesia 2018;11:41–53.
[95] Nguyen PT, Rahman MS, Le PM, et al. Trends in, projections of, and inequalities in reproductive, maternal, newborn and child health service coverage in Vietnam 2000-2030: A Bayesian analysis at national and sub-national levels. The Lancet Regional Health - Western Pacific 2021;15. https://doi.org/10.1016/j.lanwpc.2021.100230.

[96] Sharma J, Zangpo K, Grundy J. Measuring universal health coverage: a three-dimensional composite approach from Bhutan. WHO South East Asia J Public Health 2014;3:226–37. https://doi.org/10.4103/2224-3151.206745.
[97] Haakenstad A, Irvine CMS, Knight M, et al. Measuring the availability of human resources for health and its relationship to universal health coverage for 204 countries and territories from 1990 to 2019: a systematic analysis for the Global Burden of Disease Study 2019. The Lancet 2022;399:2129–54. https://doi.org/10.1016/S0140-6736(22)00532-3.
[98] Kim S, Headley TY, Tozan Y. Universal healthcare coverage and health service delivery before and during the COVID- 19 pandemic: A difference-in-difference study of childhood immunization coverage from 195 countries. PLoS Medicine 2022;19. https://doi.org/10.1371/journal.pmed.1004060.
[99] Lozano R, Fullman N, Mumford JE, et al. Measuring universal health coverage based on an index of effective coverage of health services in 204 countries and territories, 1990–2019: a systematic analysis for the Global Burden of Disease Study 2019. The Lancet 2020;396:1250–84. https://doi.org/10.1016/S0140-6736(20)30750-9.
[100] Wigley S, Dieleman JL, Templin T, et al. Autocratisation and universal health coverage: Synthetic control study. The BMJ 2020;371. https://doi.org/10.1136/bmj.m4040.
[101] Kim S, Headley TY, Tozan Y. The synergistic impact of Universal Health Coverage and Global Health Security on health service delivery during the Coronavirus Disease-19 pandemic: A difference-in-difference study of childhood immunization coverage from 192 countries. PLOS Glob Public Health 2024;4:e0003205. https://doi.org/10.1371/journal.pgph.0003205.
[102] Wang H, Song Y, Ma J, et al. Burden of non-communicable diseases among adolescents and young adults aged 10–24 years in the South-East Asia and Western Pacific regions, 1990–2019: a systematic analysis for the Global Burden of Disease Study 2019. The Lancet Child & Adolescent Health 2023;7:621–35. https://doi.org/10.1016/S2352-4642(23)00148-7.
[103] Wang C, Zheng Y, Luo Z, et al. Socioeconomic characteristics, cancer mortality, and universal health coverage: A global analysis. Med 2024;5:926-942.e3. https://doi.org/10.1016/j.medj.2024.04.002.
[104] Qin C, Liu M, Liu J. Trends and disparities of disease burden in infections among pregnant women in 131 low-income and middle-income countries, 1990–2019. J Glob Health 2024;14:04130. https://doi.org/10.7189/jogh.14.04130.
[105] Do Amaral Junior OL, Braccini Fagundes ML, Hugo FN, et al. Correlation between structural determinants and universal health coverage in 2010 and 2019: An analysis of the global burden of disease study. PLOS Glob Public Health 2025;5:e0004770. https://doi.org/10.1371/journal.pgph.0004770.
[106] Cao X, Wang M, Zhou M, et al. Global, Regional and National Burden of Paediatric Atopic Dermatitis: A Trend and Geographic Inequalities Analysis. Clin Experimental Allergy 2024;54:747–59. https://doi.org/10.1111/cea.14558.
[107] Blas L, Shiota M, Onozawa M, et al. Primary management of prostate cancer by universal health coverage effective coverage index. World J Urol 2025;43:146. https://doi.org/10.1007/s00345-025-05530-7.
[108] Dieleman JL, Sadat N, Chang AY, et al. Trends in future health financing and coverage: future health spending and universal health coverage in 188 countries, 2016–40. The Lancet 2018;391:1783–98. https://doi.org/10.1016/S0140-6736(18)30697-4.

[109] Barasa E, Nguhiu P, McIntyre D. Measuring progress towards Sustainable Development Goal 3.8 on universal health coverage in Kenya. BMJ Glob Health 2018;3:e000904. https://doi.org/10.1136/bmjgh-2018-000904.
[110] Eze P, Idemili CJ, Lawani LO. Evaluating health systems' efficiency towards universal health coverage: A data envelopment analysis. Inquiry : A Journal of Medical Care Organization, Provision and Financing 2024;61:469580241235759-. https://doi.org/10.1177/00469580241235759.
[111] Mchenga M, Manthalu G, Chingwanda A, et al. Developing Malawi's Universal Health Coverage Index. Front Health Serv 2021;1:786186. https://doi.org/10.3389/frhs.2021.786186.
[112] Wagstaff A, Neelsen S. A comprehensive assessment of universal health coverage in 111 countries: a retrospective observational study. The Lancet Global Health 2020;8:e39–49. https://doi.org/10.1016/S2214-109X(19)30463-2.
[113] Wagstaff A, Cotlear D, Eozenou PH-V, et al. Measuring progress towards universal health coverage: with an application to 24 developing countries. ECOPOL 2016;32:147–89. https://doi.org/10.1093/oxrep/grv019.
[114] Zhou Y, Li C, Wang M, et al. Universal health coverage in China: a serial national cross-sectional study of surveys from 2003 to 2018. The Lancet Public Health 2022;7:e1051–63. https://doi.org/10.1016/S2468-2667(22)00251-1.
[115] Alshehari AH, Al-Selwi AA, Agu SA, et al. Measuring progress towards universal health coverage in 22 Middle East and North African countries. Dialogues in Health 2024;5:100191. https://doi.org/10.1016/j.dialog.2024.100191.
[116] Eren Korkmaz Ö, Emecen AN. The relationship between development indicators and HIV epidemiology: a global perspective on social determinants. AIDS Care 2026:1–13. https://doi.org/10.1080/09540121.2026.2618141.
[117] Go AE, Feliciano EJG, Martinez J, et al. Global determinants of cervical cancer outcomes: a health systems analysis. Bmjonc 2026;5:e000975. https://doi.org/10.1136/bmjonc-2025-000975.
[118] Wei D, Zhang H, Li T, et al. Burden, Temporal Trends, and Future Projections of Nontraumatic Intracerebral Hemorrhage in Asia, 1990–2050: A Systematic Analysis of the Global Burden of Disease Study 2021. Neuroepidemiology 2026:1–18. https://doi.org/10.1159/000550404.
[119] Yang D, Nikoloski Z, Khalid G, et al. Pakistan's path to universal health coverage: national and regional insights. Int J Equity Health 2024;23:162. https://doi.org/10.1186/s12939-024-02232-1.
[120] Wang Y, Wang R, Jiang M, et al. Provincial inequality of China's progress towards universal health coverage: An empirical analysis in 2016–21. J Glob Health 2024;14:04122. https://doi.org/10.7189/jogh.14.04122.
[121] Hogan DR, Stevens GA, Hosseinpoor AR, et al. Monitoring universal health coverage within the Sustainable Development Goals: development and baseline data for an index of essential health services. The Lancet Global Health 2018;6:e152–68. https://doi.org/10.1016/S2214-109X(17)30472-2.
[122] World Health Organization, World Bank. Tracking Universal Health Coverage: FIrst Global Monitoring Report. Geneva: World Health Organization; 2015.

[123] World Health Organization and International Bank for Reconstruction and Development / The World Bank; 2017. Licence: CC BY-NC-SA 3.0 IGO. Tracking universal health coverage: 2017 global monitoring report. n.d.
[124] Primary Health Care on the Road to Universal Health Coverage: 2019 Monitoring Report. Executive Summary. Geneva: World Health Organization; 2021.
[125] Tracking Universal Health Coverage: 2021 Global Monitoring Report. 1st ed. Geneva: World Health Organization; 2021.
[126] Tracking Universal Health Coverage: 2023 Global Monitoring Report. 1st ed. Geneva: World Health Organization; 2023.
[127] World Bank, World Health Organization. Tracking Universal Health Coverage: 2025 Global Monitoring Report - Conference Edition. Washington, D.C.: World Bank Group; 2025.
[128] Ahmat A, Asamani JA, Abdou Illou MM, et al. Estimating the threshold of health workforce densities towards universal health coverage in Africa. BMJ Glob Health 2022;7. https://doi.org/10.1136/bmjgh-2021-008310.
[129] Day C, Gray A, Cois A, et al. Is South Africa closing the health gaps between districts? Monitoring progress towards universal health service coverage with routine facility data. BMC Health Services Research 2021;21:194. https://doi.org/10.1186/s12913-021-06171-3.
[130] Dhillon I, Jhalani M, Thamarangsi T, et al. Advancing Universal Health Coverage in the WHO South-East Asia Region with a focus on Human Resources for Health. Lancet Reg Health Southeast Asia 2023;18:100313. https://doi.org/10.1016/j.lansea.2023.100313.
[131] Eregata GT, Hailu A, Memirie ST, et al. Measuring progress towards universal health coverage: national and subnational analysis in Ethiopia. BMJ Glob Health 2019;4:e001843-. https://doi.org/10.1136/bmjgh-2019-001843.
[132] Feng XL, Zhang Y, Hu X, et al. Tracking progress towards universal health coverage for essential health services in China, 2008-2018. BMJ Glob Health 2022;7. https://doi.org/10.1136/bmjgh-2022-010552.
[133] Hone T, Gonçalves J, Seferidi P, et al. Progress towards universal health coverage and inequalities in infant mortality: an analysis of 4·1 million births from 60 low-income and middle-income countries between 2000 and 2019. The Lancet Global Health 2024;12:e744–55. https://doi.org/10.1016/S2214-109X(24)00040-8.
[134] Jordi E, Pley C, Jowett M, et al. Assessing the efficiency of countries in making progress towards universal health coverage: A data envelopment analysis of 172 countries. BMJ Global Health 2020;5. https://doi.org/10.1136/bmjgh-2020-002992.
[135] Kimario KA, Muhanga MI, Kayunze KA. Household Socio-demographic Characteristics and Progress towards Attainment of Universal Health Coverage in Kilimanjaro, Tanzania. medRxiv 2022. https://doi.org/10.1101/2022.06.18.22276172.
[136] Lee Y, Kim S, Oh J, et al. An ecological study on the association between International Health Regulations (IHR) core capacity scores and the Universal Health Coverage (UHC) service coverage index. Globalization and Health 2022;18. https://doi.org/10.1186/s12992-022-00808-6.
[137] Reid M, Gupta R, Roberts G, et al. Achieving Universal Health Coverage (UHC): Dominance analysis across 183 countries highlights importance of strengthening health workforce. PLoS ONE 2020;15. https://doi.org/10.1371/journal.pone.0229666.

[138] Takura T, Miura H. Socioeconomic Determinants of Universal Health Coverage in the Asian Region. International Journal of Environmental Research and Public Health 2022;19. https://doi.org/10.3390/ijerph19042376.
[139] Tangcharoensathien V, Hirabayashi KC, Topothai C, et al. Children and women's health in south east asia: Gap analysis and solutions. International Journal of Environmental Research and Public Health 2020;17. https://doi.org/10.3390/ijerph17103366.
[140] Folayan MO, Tantawi ME, Virtanen JI, et al. An ecological study on the association between universal health service coverage index, health expenditures, and early childhood caries. BMC Oral Health 2021;21:126. https://doi.org/10.1186/s12903-021-01500-8.
[141] Assefa Y, Assefa Gelaw Y, Endalamaw A, et al. Trend analysis and modelling of universal health coverage, Ethiopia. Bull World Health Organ 2026;104:8–16. https://doi.org/10.2471/BLT.24.292995.
[142] Chakma T, Karim S, Rabbani A. Examining the association between service coverage of UHC and global disease burden: A cross-country panel analysis. Social Science & Medicine 2025;369:117832. https://doi.org/10.1016/j.socscimed.2025.117832.
[143] Dee EC, Wu JF, Feliciano EJG, et al. National Cancer System Characteristics and Global Pan-Cancer Outcomes. JAMA Oncol 2025;11:650. https://doi.org/10.1001/jamaoncol.2025.0473.
[144] Jiang X, Xu J, Cheng F, et al. Does population density impact maternal and child health? Mediating effects of the Universal Health Coverage Service Coverage Index. BMC Public Health 2025;25:2002. https://doi.org/10.1186/s12889-025-23217-x.
[145] Lubis R, Satria FB, Rasmaliah R, et al. Impact of soil-transmitted helminths infections on anemia burden: a global analysis of children under five and reproductive-age women. BMC Public Health 2025;25:1356. https://doi.org/10.1186/s12889-025-22572-z.
[146] Muchabaiwa L, Birungi C, Sobers M, et al. Universal Health Service Coverage under threat? A study of the effects of PEPFAR HIV funding on UHC. African Journal of AIDS Research 2025;24:87–96. https://doi.org/10.2989/16085906.2025.2577377.
[147] Rai A, Khatri RB, Assefa Y. Primary Health Care Systems and Their Contribution to Universal Health Coverage and Improved Health Status in Seven Countries: An Explanatory Mixed-Methods Review. IJERPH 2024;21:1601. https://doi.org/10.3390/ijerph21121601.
[148] Raza Khan M, Mohammad KU, Rabbani MS. Impact of health expenditure on universal health coverage (UHC) (composite index): Global evidence. Health Promot Perspect 2025;15:268–77. https://doi.org/10.34172/hpp.025.43192.
[149] Wang M, Song S, Jin Y, et al. Role of universal health coverage in improving quality of breast cancer care: an international comparison study. Bmjph 2024;2:e000863. https://doi.org/10.1136/bmjph-2023-000863.
[150] Zhou M, Jiang Y, Zhu J, et al. Cross-national disparities in non-communicable disease: a universal health coverage-based service coverage index perspective, 2000-2021. Front Public Health 2026;14:1756485. https://doi.org/10.3389/fpubh.2026.1756485.
[151] Zaka N, Umar M, Ahmad AM, et al. Equity trends for the UHC service coverage sub-index for reproductive, maternal, newborn and child health in Pakistan: evidence from demographic health surveys. International Journal for Equity in Health 2023;22. https://doi.org/10.1186/s12939-023-02043-w.

[152] Mafiana JJ, Shen X, Hu W, et al. Insight into Nigeria's progress towards the universal coverage of reproductive, maternal, newborn and child health services: a secondary data analysis. BMJ Open 2022;12. https://doi.org/10.1136/bmjopen-2022-061595.
[153] Mukherji A, Rao M, Desai S, et al. District-level monitoring of universal health coverage, India. Bull World Health Organ 2024;102:630-638B. https://doi.org/10.2471/BLT.23.290854.
[154] Nandi S, Schneider H, Garg S. Assessing geographical inequity in availability of hospital services under the state-funded universal health insurance scheme in Chhattisgarh state, India, using a composite vulnerability index. Global Health Action 2018;11:1541220. https://doi.org/10.1080/16549716.2018.1541220.
