# Supplementary material for: Measuring multisector nutrition and health intervention coverage using composite coverage analysis methods: a scoping review and methodological guidance
Source: BMJ Open. 2026 Jun 17;16(6):e111298. doi: 10.1136/bmjopen-2025-111298 (PMC13288883; doi:10.1136/bmjopen-2025-111298)
Supplement: Supplementary file 3 [file bmjopen-2025-111298.reviewer_comments.pdf]

PEER REVIEW HISTORY

BMJ Open publishes all reviews undertaken for accepted manuscripts. Reviewers are asked to complete a checklist review form and are provided with free text boxes to elaborate on their assessment. These free text comments are reproduced below.

ARTICLE DETAILS

Title (Provisional)

Measuring multi-sector nutrition and health intervention coverage using composite coverage analysis methods: A scoping review and methodological guidance

Authors

Morrison, Taylor; Akseer, Nadia; Heidkamp, Rebecca; Maïga, Abdoulaye; Tasic, Hana

---

VERSION 1 - REVIEW

**Reviewer**	**1**
**Name**	**Nagashima, Yasmin Guerreiro**
**Affiliation**	**Federal University of Rio Grande do Norte**
**Date**	**02-Nov-2025**
**COI**	**None**

The manuscript is organized and written clearly. The discussion is comprehensive and consistent with the study objectives. Overall, this is a strong and well-executed study that contributes meaningful evidence to the field. Unfortunately, it was developed only by one reviewer.

**Reviewer**	**2**
**Name**	**Bennett, Charles L**
**Affiliation**	**University of South Carolina**
**Date**	**08-Dec-2025**
**COI**	**None**

To the authors:

Title: The paper is a scoping review—could the authors provide some words in the text that might help the reader anticipate some of the findings---

Abstract

Introduction—why do composite indices provide such an opportunity – i.e. is there something wrong with individual markers and therefore a need for a composite index? A statement about why individual measures do not capture the totality of nutrition and health interventions would be helpful.

Methods: was there any interrater reliability measurement?

Results: these are nicely written

Conclusion: Is the gap the finding that more than a third have not been validated?

Manuscript:

Messages:

The first sentence is strong—it should have been included in the abstract's introduction.

Second message—what are the gaps that were identified?

How this study might be used-

Should some of these indices be used going forward?

Introduction:

Please define SCGs. Please define Examplars.

Is there a need for standard approaches? Should approaches be somewhat context specific? For example, LICs and LMICs might use different approaches. Can the authors provide references for composite measures in economics and social development literature?

In this scoping review, why not evaluate measures according to LICs, LMICs etc rather than one size should fit all.

Methods:

Why not have the search done by two authors and inter-rater reliability be measured?

Results:

Can you break out the indices according to LICs. LMICs, and other—and report on the findings by these groupings?

Discussion

How much of the study results are from the grey literature? Would the study findings be different if no grey literature were included.

Is there a conceptual framework that can be derived based on the literature review?

Can the authors provide some insight into how expensive and time consuming.

Reviewer 3

**Name**	**Akter, Fahmida**
**Affiliation**	**BRAC University James P Grant School of Public Health**
**Date**	**16-Dec-2025**
**COI**	**None**

Manuscript ID: bmjopen-2025-111298

Thank you for the opportunity to review this excellent article. The findings are important to address a clear evidence gap by measuring multi-sector nutrition and health intervention coverage using composite coverage analysis methods. The manuscript is well written, and I enjoyed reading it. However, I have a few concerns regarding clarity, explanation, and formatting, as outlined below:

Abstract

• The abstract does not fully adhere to the prescribed abstract format required by BMJ Open (https://bmjopen.bmj.com/pages/authors). Please revise the whole abstract to align with the journal's guideline.

Strength and limitation of this study

• Instead of the 'key messages' section, the authors should include a section titled 'Strengths and limitations of this study', as per authors guideline (https://bmjopen.bmj.com/pages/authors). This section should be no more than 5 bullet points relating specifically to the methods - not the results of the study. This will be published as a summary box after the abstract in the final published article.]

Methods

• Line 152-154: 'Measures of disease burdens and health impacts, such as health-adjusted life years (HALYs), quality-adjusted life years (QALYs), and disability-adjusted life years (DALYs), were excluded'. While the exclusion is understandable, please add a brief explanatory sentence clarifying why HALYs, QALYs, and DALYs were excluded from the analysis.

• Line 155-156: '…related to maternal and child health nutrition coverage used in LMICs were prioritized'. The meaning of 'prioritized' is unclear. Please elaborate with one or two sentences describing how these were prioritized, what actions were taken, and what the implications were.

• Line 159: '…between 1980 and May 18, 2024, were considered'. Please add a short justification for selecting 1980 as the starting year.

• Line 165-166: 'The reviewer (TM) resolved uncertainties by discussion with the co-authors'. Please include the initials of the co-authors involved in these discussions.

• For ease of reading, please repeat the header row in Table 1 on subsequent pages.

VERSION 1 - AUTHOR RESPONSE

Reviewer: 1

Dr. Yasmin Guerreiro Nagashima, Federal University of Rio Grande do Norte
Comments to the Author:
(1) The manuscript is organized and written clearly. The discussion is comprehensive and consistent with the study objectives. Overall, this is a strong and well-executed study that contributes meaningful evidence to the field. Unfortunately, it was developed only by one reviewer.

Response:
We acknowledge the limitation of having one reviewer conduct the screening and data extraction. We have stated this limitation in the strengths and limitations section of the manuscript. This was a smaller project that required a rapid review of a niche topic. To account for the limitation, the single researcher meticulously documented their search strategy and inclusion/exclusion criteria, verifying each step with the other authors (NA and RH). Finally, as this is a scoping review, not a systematic review, we understand that having one reviewer can be acceptable under the above conditions.

Reviewer: 2

Dr. Charles L Bennett, University of South Carolina
Comments to the Author:
To the authors:

(1) Title: The paper is a scoping review—could the authors provide some words in the text that might help the reader anticipate some of the findings---

Response:
We have revised the title of the paper to allude to the conclusions and methodological guidance that were the conclusion of this scoping review.

Abstract
(2) Introduction—why do composite indices provide such an opportunity – i.e. is there something wrong with individual markers and therefore a need for a composite index? A statement about why individual measures do not capture the totality of nutrition and health interventions would be helpful.

Response:
We have added a statement about why composite measures provide a unique opportunity over individual measures of health and nutrition intervention coverage on line 34 of the abstract.

(3) Methods: was there any interrater reliability measurement?

Response:
No, there was no interrater reliability measurement because there was only one reviewer conducting screening and data extraction. This is mentioned in the methods and strengths and limitation sections of the manuscript.

(4) Conclusion: Is the gap the finding that more than a third have not been validated?

<u>Response:</u>
There are two primary gaps identified in this study. The first is the fact that to our knowledge there are no multisectoral nutrition composite indices currently developed or in use. The second is the fact that more than a third of the identified indices related to nutrition have not been validated. These are described in the conclusion of the abstract.

We have added a sentence to clarify the main conclusion of the scoping review on line 512 of the conclusion: “To our knowledge there are no multisectoral nutrition composite indices currently developed or in use and more than a third of the composite indices currently in use for measuring coverage of multiple health and/or nutrition interventions or health and nutritional status have not been validated.”

Manuscript
(5) Messages:
The first sentence is strong—it should have been included in the abstract’s introduction.

<u>Response:</u>
The first sentence of the first message has been included in the abstract’s objectives on line 37: “Of the many composite indices in use to measure health and nutrition intervention coverage and population status as a means of assessing progress in achieving health and nutrition targets, few have been previously documented in the nutrition and health literature, nor have these indicators been described in detail.”

(6) Second message—what are the gaps that were identified?
How this study might be used-
Should some of these indices be used going forward?

<u>Response:</u>
The second message has been removed as it does not comply with the journal’s submission guidelines.
We have added a section about how this study may be used in the discussion, which we suggest can be used as a reference for the use of nutrition and health composite indices. We also suggest the review be used as a guide for best practices in composite index design and validation (Line 391).

(7) Introduction:
Please define SCGs. Please define Exemplars.
Is there a need for standard approaches? Should approaches be somewhat context specific? For example, LICs and LMICs might use different approaches. Can the authors provide references for composite measures in economics and social development literature?
In this scoping review, why not evaluate measures according to LICs, LMICs etc rather than one size should fit all.

<u>Responses:</u>
- We have added a sentence defining SDGs (Line 106)
- We have added a sentence defining Exemplars (Line 108)

- We advocate for a standard guide for index development that is flexible so that indices can be designed to be fit to purpose (Line 144). Certainly, different contexts will require different approaches just as different contexts require different combinations of interventions.
- We added a reference for the Human Development Index (HDI) as a composite measure used in economics and social development (Line 138)
- We intended to assess the methodologies used in the development of composite indices in nutrition and health instead of the application of such indices. The main results we were aiming to assess included the methods of selecting indicators, aggregating indicators, and validating composite indices. These methods are applicable to composite index development and use independent of the context in which you wish to measure composite coverage.

(8) Methods:
Why not have the search done by two authors and inter-rater reliability be measured?

Response:
We acknowledge the limitation of having one reviewer conduct the screening and data extraction. We have stated this limitation in the strengths and limitations section of the manuscript. This was a smaller project that required a rapid review of a niche topic. To account for the limitation, the single researcher meticulously documented their search strategy and inclusion/exclusion criteria, verifying each step with the other authors (NA and RH). Finally, as this is a scoping review, not a systematic review, we understand that having one reviewer can be acceptable under the above conditions.

There was no interrater reliability measurement because there was only one reviewer conducting screening and data extraction. This is mentioned in the methods and strengths and limitation sections of the manuscript.

(9) Results:
Can you break out the indices according to LICs. LMICs, and other—and report on the findings by these groupings?

Response:
This is intended to be a review of global index methodologies and a source of information on the methods used, not the settings in which these indices have been used. Therefore, we do not think it is informative to organize the indices by application in World Bank Classification of Low- and Middle-Income Countries. However, we have included the geographical application of each of the indices as some indices are specific to certain country contexts based on the indicators used in the measurement of the composite index.

(10) Discussion
How much of the study results are from the grey literature? Would the study findings be different if no grey literature were included.
Is there a conceptual framework that can be derived based on the literature review?
Can the authors provide some insight into how expensive and time consuming.

Response:

Details on the number of grey literature records and the composition of those grey literature sources have been added to lines 251 and 264. The study findings are not significantly different with grey literature excluded.
Yes, a step-by-step framework is being developed based on this scoping review. A manuscript is being written as a separate piece of work, and we have mentioned that this is underway in the discussion. This follow-up manuscript will contain detail on the time and cost of conducting such an exercise.

Reviewer: 3

Dr. Fahmida Akter, BRAC University James P Grant School of Public Health
Comments to the Author:
Manuscript ID: bmjopen-2025-111298
Thank you for the opportunity to review this excellent article. The findings are important to address a clear evidence gap by measuring multi-sector nutrition and health intervention coverage using composite coverage analysis methods. The manuscript is well written, and I enjoyed reading it. However, I have a few concerns regarding clarity, explanation, and formatting, as outlined below:

Abstract
(1) The abstract does not fully adhere to the prescribed abstract format required by BMJ Open (https://bmjopen.bmj.com/pages/authors). Please revise the whole abstract to align with the journal's guideline.

Response:
The abstract has been revised to include the following sections on line 32:

- Objectives
- Design
- Data sources
- Eligibility criteria
- Data extraction and synthesis
- Results
- Conclusions

(2) Strength and limitation of this study
Instead of the 'key messages' section, the authors should include a section titled 'Strengths and limitations of this study', as per authors guideline (https://bmjopen.bmj.com/pages/authors). This section should be no more than 5 bullet points relating specifically to the methods - not the results of the study. This will be published as a summary box after the abstract in the final published article.]

Response:
The following sections have been deleted: What is already known on this topic; What this study adds; and How this study might affect research, practice or policy. A section entitled 'Strengths and limitations of this study' has been added immediately after the abstract and contains 3 short bullet points that relate to the methods of the scoping review.

(3) Methods

Line 152-154: ‘Measures of disease burdens and health impacts, such as health-adjusted life years (HALYs), quality-adjusted life years (QALYs), and disability-adjusted life years (DALYs), were excluded’. While the exclusion is understandable, please add a brief explanatory sentence clarifying why HALYs, QALYs, and DALYs were excluded from the analysis.

Response:
An explanatory sentence clarifying why HALYs, QALYs, and DALYs were excluded was added to line 187.

Line 155-156: ‘…related to maternal and child health nutrition coverage used in LMICs were prioritized’. The meaning of ‘prioritized’ is unclear. Please elaborate with one or two sentences describing how these were prioritized, what actions were taken, and what the implications were.

Response:
We have removed the sentence: ‘…related to maternal and child health nutrition coverage used in LMICs were prioritized’ because this paper had no empirical way of prioritizing certain composite measures. Instead, the team plans to utilize the findings specific to maternal and child health nutrition coverage for future research (Line 189).

Line 159: ‘…between 1980 and May 18, 2024, were considered’. Please add a short justification for selecting 1980 as the starting year.

Response:
Justification for the limit of 1980 is due to our original inclusion of indices such as the Human Development Index (HDI), which was introduced in 1990. We therefore assumed that there could be methodological papers published between 1980 and 1990 that described the development of such foundational indices. A statement has been added to line 194 in the Main Document – marked copy.

Line 165-166: ‘The reviewer (TM) resolved uncertainties by discussion with the co-authors’. Please include the initials of the co-authors involved in these discussions.

Response:
We have added the initials of the co-authors involved in discussing uncertainties (Line 206).

For ease of reading, please repeat the header row in Table 1 on subsequent pages.
The header row in Table 1 has been repeated on subsequent pages for ease of reading.

Response:
The header row in Table 1 has been repeated on subsequent pages for ease of reading.

VERSION 2 - REVIEW

Reviewer 2

**Name**	**Bennett, Charles L**
**Affiliation**	**University of South Carolina**
**Date**	**24-Apr-2026**
**COI**	

Outstanding revision!!1 Great job...

**Reviewer**	**3**
**Name**	**Akter, Fahmida**
**Affiliation**	**BRAC University James P Grant School of Public Health**
**Date**	**04-Apr-2026**
**COI**	

Manuscript ID: bmjopen-2025-111298.R1

The authors have carefully addressed all my comments, and the manuscript has been substantially improved. I am satisfied with the revisions and recommend acceptance. Congratulations to the authors on this work.
